# Prox1-positive cells monitor and sustain the murine intestinal epithelial cholinergic niche

**DOI:** 10.1038/s41467-019-13850-7

**Published:** 2020-01-08

**Authors:** Moritz Middelhoff, Henrik Nienhüser, Giovanni Valenti, H. Carlo Maurer, Yoku Hayakawa, Ryota Takahashi, Woosook Kim, Zhengyu Jiang, Ermanno Malagola, Krystle Cuti, Yagnesh Tailor, Leah B. Zamechek, Bernhard W. Renz, Michael Quante, Kelley S. Yan, Timothy C. Wang

**Affiliations:** 1 Division of Digestive and Liver Diseases, Department of Medicine, Columbia University Medical Center, New York, NY 10032 USA; 2 Klinikum rechts der Isar, II. Medizinische Klinik, Technische Universität München, 81675 Munich, Germany; 3 Graduate School of Medicine, Department of Gastroenterology, The University of Tokyo, Tokyo, 113-0033 Japan; 4 Klinik für Allgemein-, Viszeral- und Transplantationschirurgie, Ludwig-Maximilians-Universität München, 81377 Munich, Germany; 5 Department of Genetics and Development, Columbia University Medical Center, New York, NY 10032 USA

**Keywords:** Extracellular signalling molecules, Neural circuits, Intestinal stem cells, Stem-cell differentiation, Stem-cell niche

## Abstract

The enteric neurotransmitter acetylcholine governs important intestinal epithelial secretory and immune functions through its actions on epithelial muscarinic Gq-coupled receptors such as M3R. Its role in the regulation of intestinal stem cell function and differentiation, however, has not been clarified. Here, we find that nonselective muscarinic receptor antagonism in mice as well as epithelial-specific ablation of M3R induces a selective expansion of DCLK1-positive tuft cells, suggesting a model of feedback inhibition. Cholinergic blockade reduces Lgr5-positive intestinal stem cell tracing and cell number. In contrast, Prox1-positive endocrine cells appear as primary sensors of cholinergic blockade inducing the expansion of tuft cells, which adopt an enteroendocrine phenotype and contribute to increased mucosal levels of acetylcholine. This compensatory mechanism is lost with acute irradiation injury, resulting in a paucity of tuft cells and acetylcholine production. Thus, enteroendocrine tuft cells appear essential to maintain epithelial homeostasis following modifications of the cholinergic intestinal niche.

## Abstract (web summary)

Acetylcholine regulates intestinal epithelial secretion via muscarinic Gq-coupled receptors but its role in cell differentiation is unclear. Here, the authors show that Prox1-positive endocrine cells are sensors for the cholinergic intestinal niche and can trigger increased differentiation of enteroendocrine DCLK1-positive tuft cells.

## Introduction

The activity of intestinal crypt stem cells (ISC) is governed by complex signals from the surrounding niche^1^. The enteric nervous system is considered part of this niche^2^, and acetylcholine (Ach), as the most predominant enteric neurotransmitter^3^, exerts its functions by acting on nicotinic or muscarinic receptors on target cells^4^. Enteric neurons modulate epithelial proliferation^5^ via muscarinic receptors^6,7^ and muscarinic signaling has been shown to act on intracellular pathways, such as Wnt^8^ or *trans*-activate EGFR^9^. However, the influence of cholinergic signaling on ISC activity and differentiation remains to be clarified.

In the intestinal epithelium, the expression of the rate-limiting enzyme for the production of Ach, Choline acetyltransferase (ChAT), is unique to tuft cells^10^. Intestinal tuft cells comprise a heterogeneous cell lineage^11^ and have been divided into an immune and neuronal phenotype^12,13^. While important immune modulatory functions have recently emerged^14–17^, the functional relevance of the proposed neuronal tuft phenotype remains elusive. In addition, tuft cells have been proposed to be an important epithelial component of the ISC niche, as the loss of tuft cells appears to impair intestinal regeneration following injury^18^. The cellular origin of tuft cells has been attributed to Lgr5-positive ISC in the crypt base^19^, and the development of the secretory, immune phenotype appears to depend on the expression of *Sox4*^20^. However, previous work by Bjerknes and Cheng argued for the existence of tuft cell progenitors located just above the crypt base at cell positions +4 to +5^21^.

In line with this observation, Prox1-positive endocrine cells with progenitor features located above the crypt base appeared to be closely related to the tuft cell lineage, supporting a common origin of endocrine and tuft cells^22^. Here, we show that disruption of muscarinic cholinergic signaling reduces Lgr5-positive stem cell tracing and number. Concomitantly, Prox1-positive cells appear as the primary sensors of cholinergic interruption, as they orchestrate the expansion and differentiation of progenitors into an enteroendocrine tuft cell phenotype. These enteroendocrine tuft cells upregulate important signaling pathways to sustain epithelial homeostasis, which is in part orchestrated by an increase of mucosal Ach release, suggestive of a compensatory response circuit to maintain epithelial cholinergic input.

## Results

### Muscarinic receptor blockade induces tuft cell expansion

**Fig. 1:**
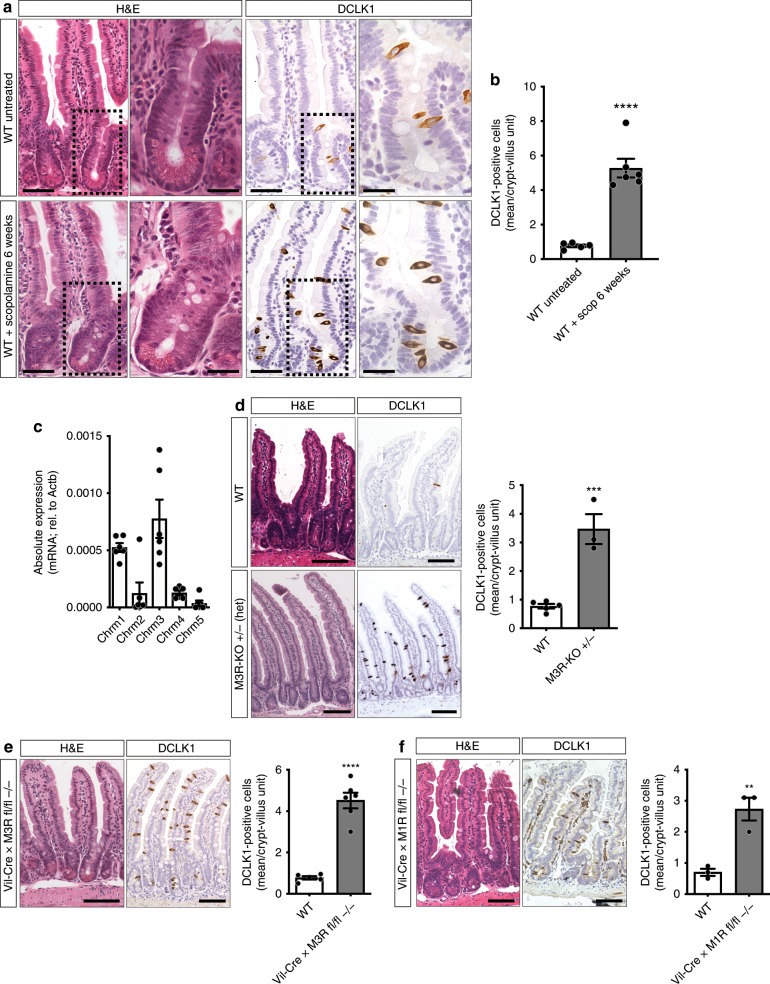
Epithelial sensing of muscarinic receptor blockade results in selective DCLK1-positive tuft cell expansion. **a**, **b** Histologic analysis of WT (untreated) and scopolamine-treated WT (6 weeks) murine jejunal tissues. Scopolamine treatment provoked selective expansion of DCLK1-positive tuft cells (WT untreated (*n* = 5), Mean = 0.77, SEM = 0.080; WT + scopolamine 6 weeks (*n* = 6), Mean = 5.281, SEM = 0.543; unpaired *t* test, two-tailed, *t* = 7.453, df = 9); bar graphs left H&E, DCLK1 = 50 µm, magnifications H&E, DCLK1 right = 25 µm. **c** mRNA analysis of epithelial-enriched jejunal preparations for muscarinic receptors M1R—M5R (*Chrm1*—*Chrm5*) showed predominant expression of M3R and M1R (*n* = 6 WT mice). **d** Analysis of heterozygous M3R-KO mice (whole body-KO) showed significant DCLK1-positive tuft cell expansion (*n* = 5 WT mice, Mean = 0.77, SEM = 0.080; *n* = 3 M3R-KO mice, Mean = 3.467, SEM = 0.524; unpaired *t* test, two-tailed, *t* = 6.790, df = 6); bar graphs = 100 µm. **e** Tuft cell expansion similarly resulted from epithelial ablation of M3R employing Vil-Cre × M3R fl/fl mice (*n* = 5 WT mice, Mean = 0.77, SEM = 0.080; *n* = 6 Vil-Cre × M3R fl/fl −/− mice, Mean = 4.517, SEM = 0.377; unpaired *t* test, two-tailed, *t* = 8.848, df = 9); bar graphs = 100 µm. **f** Epithelial ablation of M1R employing Vil-Cre × M1R fl/fl mice resulted in modest DCLK1-positive tuft cell expansion (*n* = 3 mice each group, WT Mean = 0.7, SEM = 0.115; Vil-Cre × M1R fl/fl −/− Mean = 2.733, SEM = 0.367; unpaired *t* test, two-tailed, *t* = 5.280, df = 4); bar graphs = 100 µm. Source data are provided as a Source Data file. **p < 0.01, ***p < 0.005, ****p < 0.001.

Muscarinic receptors (M1R—M5R) signal via G_αq/11_ or G_i/o_ and govern mucosal ion transport^23^, epithelial proliferation^7^, and barrier function^24^ or immune host defense mechanisms^8^. Taking into consideration the reported turnover times for intestinal Paneth and endocrine cell types^25,26^, long-term treatment of WT mice with the nonselective muscarinic receptor antagonist scopolamine^7^ was employed to investigate the role of muscarinic signaling on the intestinal epithelial cell differentiation. At baseline, scopolamine treatment did not affect intestinal crypt-villus architecture in regards to villus height or crypt depth (Supplementary Fig. 1A). When looking at differentiated cell types, we observed an unexpected, robust, and selective expansion of DCLK1-positive tuft cells (>6.5-fold) (Fig. 1a, b). In addition to DCLK1 immunostaining, tuft cells were identified morphologically^27^. In contrast, no significant changes in cell numbers were observed for goblet (Alcian blue), Paneth (Lysozyme 1), and enterochromaffin (ChgA) cell numbers (Supplementary Fig. 1B). Of note, already after 7 days of scopolamine treatment we could observe a moderate expansion of DCLK1-positive tuft cells (Supplementary Fig. 1C).

In line with the importance of M3R for intestinal homeostasis^24^, we found that the expression of *Chrm3* (the gene coding for M3R) in intestinal epithelial-enriched WT samples was the highest among cholinergic receptors, followed by *Chrm1* (the gene coding for M1R) (Fig. 1c). Subsequently, we observed a similar selective expansion (4.5-fold) of DCLK1-positive tuft cells in mice heterozygous for the constitutive (whole body) knockout of the M3 receptor compared with WT mice (M3R-KO, Fig. 1d). *Chrm3* expression levels were significantly reduced in these mice (Supplementary Fig. 1D). Homozygous M3R-KO, however, were difficult to breed and demonstrated increased mortality at 6–8 weeks of age. In contrast, whole body homozygous M1R-KO mice bred well, and also demonstrated a pronounced tuft expansion, although to a lesser extent than M3R-KO (Supplementary Fig. 1E).

Next, we tested whether the disruption of cholinergic signaling was primarily sensed by intestinal epithelial cells. Vil-Cre × M3R fl/fl mice were employed to conditionally ablate M3R in intestinal epithelial cells. In these conditional knockout mice, tuft cells indeed expanded similarly to that seen in M3R-KO mice (greater than fivefold; Fig. 1e), and RT-PCR analysis of epithelial-enriched samples from Vil-Cre × M3R fl/fl mice confirmed the complete loss of *Chrm3* (Supplementary Fig. 2A). These results indicate the presence of epithelial sensing of cholinergic signaling disruption in the intestine, and confirmed that the expansion was specific to DCLK1-positive tuft cells, as the numbers of closely related endocrine PYY- and ChgA-positive cell types (Supplementary Fig. 2B, C), along with secretory-, endocrine-, or enterocyte-related mRNA transcripts (Supplementary Fig. 2D), remained unchanged.

In line with the lower levels of *Chrm1* intestinal expression, epithelial ablation of M1R in Vil-Cre × M1R fl/fl mice also led to an expansion of tuft cells, although the change was more modest compared with that observed with epithelial M3R ablation (Fig. 1f). To test whether M3R and M1R are indeed both important in governing epithelial cholinergic transmission, we generated Vil-Cre × M3R fl/fl × M1R fl/fl mice (double-KO), which showed an additive effect (Supplementary Fig. 2E) compared with ablation of M3R alone, resulting in a dramatic greater than ninefold tuft expansion in the double-KO compared with WT tissues. Histologic analysis of Vil-Cre × M3R fl/fl × M1R fl/fl mice, and, to a lesser extent, scopolamine-treated mice, showed enlarged goblet cells while Paneth cells appeared misplaced in the upper crypt, reminiscent of the appearance of intermediate cells following G_αq/11_ perturbations in earlier studies^28^ (Supplementary Figs. 1B and 2E, white arrowheads).

### Prox1-positive cells primarily orchestrate tuft expansion

**Fig. 2:**
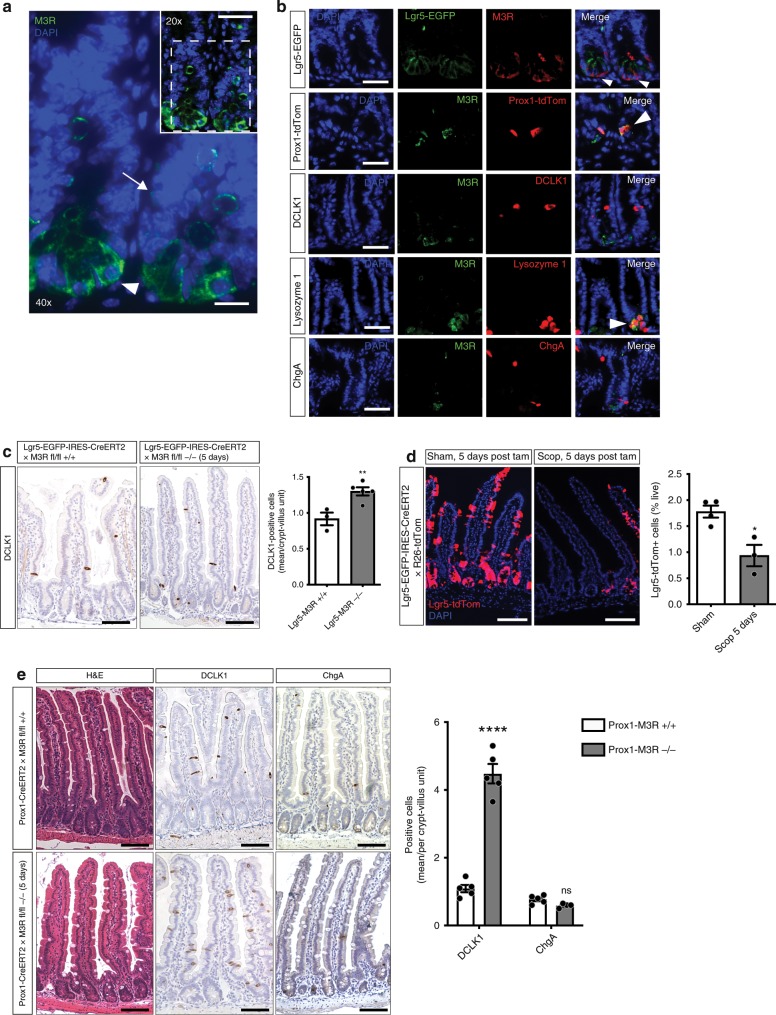
Muscarinic receptor blockade reduces Lgr5-positive ISC tracing and sensing Prox1-positive endocrine cells primarily orchestrate tuft expansion. **a** Immunostainings for M3R showed distribution of the receptor in the crypt base cell compartment (white arrowhead) as well as in cells in positions +4 to +5 of the crypt (white arrow); bar graph top = 50 µm; magnification = 25 µm. **b** Representative pictures of co-stainings of M3R with intestinal tissue from Lgr5-EGFP-IRES-CreERT2 and induced Prox1-CreERT2 × R26-tdTom mice, respectively, and WT tissue stained for DCLK1, Lysozyme 1, or ChgA (the stainings were repeated at least twice per antibody; positive overlap indicated with white arrowheads); bar graphs = 50 µm. **c** DCLK1-positive tuft cell frequency within the Lgr5 lineage following M3R ablation in Lgr5-EGFP-IRES-CreERT2 × M3R fl/fl mice 5 days after induction (*n* = 3 Lgr5-EGFP-IRES-CreERT2 × M3R fl/fl +/+ mice, Mean = 0.917, SEM = 0.088; *n* = 5 Lgr5-EGFP-IRES-CreERT2 × M3R fl/fl −/− mice, Mean = 1.3, SEM = 0.057; unpaired *t* test, two-tailed, *t* = 3.848, df = 6); bar graphs = 100 µm. **d** Lgr5-positive ISC tracing is reduced following nonselective muscarinic receptor antagonism (*n* = 4 mice sham, Mean = 1.78, SEM = 0.1151; *n* = 3 mice scopolamine, Mean = 0.9367, SEM = 0.2050; unpaired *t* test, two-tailed, *t* = 3.851, df = 5); bar graphs = 100 µm. **e** In contrast, ablation of M3R in Prox1-positive endocrine cells resulted in robust DCLK1-positive tuft cell expansions 5 days after induction of Prox1-CreERT2 × M3R fl/fl mice (*n* = 5 Prox1-CreERT2 × M3R fl/fl +/+ and M3R fl/fl −/− mice for DCLK1, *n* = 5 Prox1-CreERT2 × M3R fl/fl +/+ mice, *n* = 4 Prox1-CreERT2 × M3R fl/fl−/− for ChgA; Prox1-CreERT2 × M3R fl/fl +/+ DCLK1 Mean = 1.090, SEM = 0.109; ChgA Mean = 0.77, SEM = 0.054; Prox1-CreERT2 × M3R fl/fl −/− DCLK1 Mean = 4.480, SEM = 0.286, ChgA Mean = 0.588, SEM = 0.031; ordinary two-way ANOVA, DCLK1 *t* = 14.90, df = 15; ChgA *t* = 0.7565, df = 15); bar graphs = 100 µm. Source data are provided as a Source Data file. *p < 0.05, **p < 0.01, ****p < 0.001, ns not significant.

The M3R is believed to be expressed in intestinal stem cells (ISC) at the crypt base^6^, but the precise sites of M3R expression in the crypt epithelium remain unclear. Thus, to identify the potential cell type(s) responsible for sensing levels of cholinergic signaling, immunostaining for M3R was performed. These studies demonstrated M3R expression in numerous cells at the crypt base, as well as cells in the +4 to +5 cell positions (Fig. 2a). The M3R-positive crypt base cells resembled Lgr5-positive ISC, and co-staining in Lgr5-EGFP-CreERT mice indeed demonstrated good overlap (Fig. 2b). Endocrine cell types with progenitor features have recently been identified in cell positions +4/+5 of the crypt^22^, and we could detect prominent M3R co-staining with Prox1-positive endocrine cells (Fig. 2b). Additional immunostaining also confirmed the presence of M3R in Lysozyme-positive Paneth cells, while we were unable to detect the presence of M3R in DCLK1-positive tuft or ChgA-positive enterochromaffin cells.

This was confirmed by analysis of recently published single-cell RNA-sequencing data from intestinal epithelial cells^13^, which showed the highest level of *Chrm3* expression in secretory cells (Paneth, goblet cells), followed by the stem and endocrine cell compartments (Supplementary Fig. 3A). In this dataset, the stem compartment was primarily defined by expression of stem cell markers, such as *Lgr5*, *Ascl2*, *Axin2* or *Olfm4*, whereas the endocrine cell compartment was defined by expression of markers, such as *ChgA*, *Tac1*, *Neurog3* or *Prox1*. Only negligible expression of *Chrm3* was detected in tuft cells. Thus, while Ach signaling in Paneth and goblet cells is thought to drive luminal secretion^29,30^, Lgr5-positive ISC and Prox1-positive endocrine cells appear able to respond to alterations in cholinergic signal and thus are potential candidates for the cholinergic sensor cells.

Despite very low *Chrm3* expression in tuft cells, we first analyzed the effects of muscarinic antagonism on traced tuft cells, in induced Dclk1-BAC-CreERT × R26-tdTom mice treated with scopolamine for 6 weeks. This treatment did not result in an expansion of epithelial Dclk1-tdTom-positive tuft cells (Supplementary Fig. 3B), indicating that existing, mature tuft cells do not give rise to new tuft cells in response to reduced cholinergic signaling. Thus, the expanding DCLK1-positive tuft cells must be newly generated, which likely involves sensing by progenitors such as Lgr5-positive ISC^19^, or closely related Prox1-positive endocrine cells^22^.

Indeed, short-term ablation of M3R in the Lgr5 lineage did result in a modest expansion of DCLK1-positive tuft cells (Fig. 2c), suggesting a portion of *Lgr5*-expressing cell types or their early descendants sense M3R ablation to give rise to tuft cells. Simultaneously, however, broad muscarinic receptor blockade employing scopolamine treatment significantly reduced Lgr5-positive cell lineage tracing (Fig. 2d and Supplementary Fig. 3C, D). Furthermore, the Lgr5-EGFP-positive cell pool declined following scopolamine treatment of Lgr5-EGFP-DTR mice for 7 days (Supplementary Fig. 3E), indicating that disruption of cholinergic signaling reduces both the number and activity of Lgr5-positive ISC.

In contrast to these findings regarding Lgr5, analysis of intestinal tissues from short-term tamoxifen-induced Prox1-CreERT2 × M3R fl/fl mice showed a robust tuft expansion (greater than fourfold; Fig. 2e). This increase was notable, given that the tuft expansion observed appeared similar in degree to the increase seen with constitutive ablation of M3R in Vil-Cre × M3R fl/fl mice (Fig. 1e). In addition, this phenotype persisted for up to 6 weeks following induction (Supplementary Fig. 3F), in line with the previously suggested longevity of intestinal Prox1-positive cells^22^. In line with Vil-Cre × M3R fl/fl tissues, ChgA-positive endocrine cells did not change following M3R ablation in Prox1-positive cells (Fig. 2e). Prox1-positive cells were distinct from Lgr5-positive ISCs, with no observable overlap in induced Prox1-CreERT2 × R26-tdTom × Lgr5-EGFP-DTR mice (Supplementary Fig. 3G). Unexpectedly, Prox1-positive cell tracing did not change significantly following ablation of M3R in the Prox1 lineage or muscarinic antagonism, with an absence of Prox1 tracing of expanding tuft cells (Supplementary Fig. 3H, I).

### Expanding tuft cells adopt enteroendocrine differentiation

Tuft cells have been classified into an immune/inflammatory and neuronal phenotype^12,13^, and immune tuft cell expansion has been demonstrated to be essential in orchestrating intestinal helminth clearance by inducing a type 2 immune response^14,15^. Whole body M3R-KO mice show a significantly abolished type 2 immune response to intestinal helminths^24^, suggesting that cholinergic modulations may indeed induce a functionally distinct tuft phenotype. Thus, to test whether muscarinic receptor blockade induces a functionally distinct tuft cell phenotype, recently generated BAC transgenic Dclk1-DTR-ZSgreen mice^31^ (see “Methods” section for details) were treated with scopolamine for 7 days and sorted ZSgreen-positive tuft cells subjected to bulk RNA-sequencing analysis (Supplementary Fig. 4A).

**Fig. 3:**
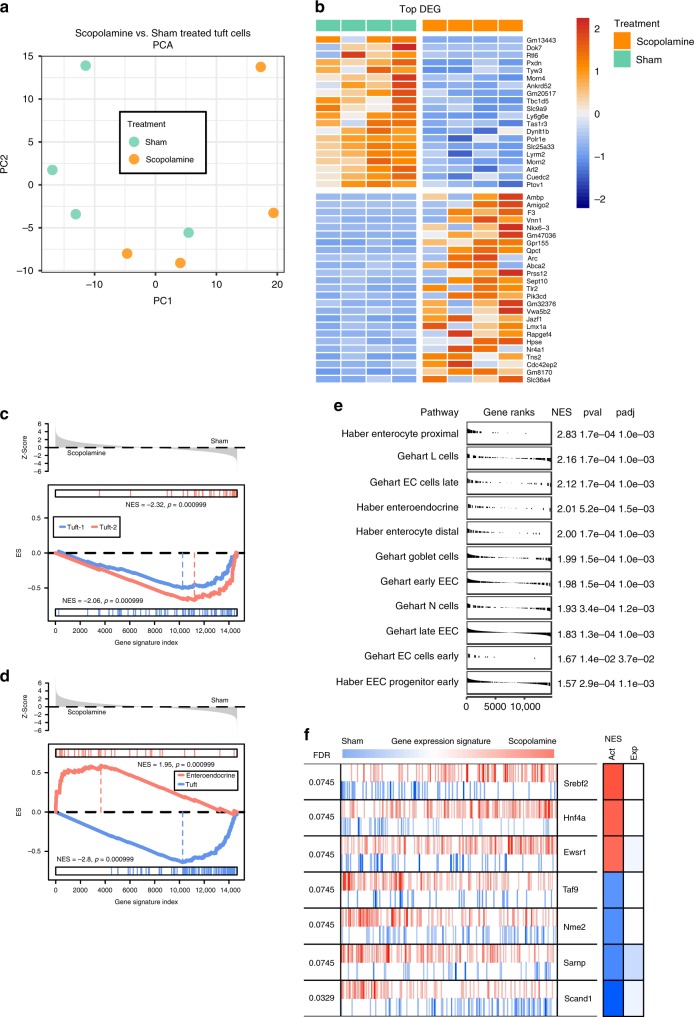
RNA-sequencing analysis indicates adoption of enteroendocrine differentiation of expanding tuft cells following scopolamine treatment. **a** Scopolamine-treated Dclk1-DTR-ZSgreen-positive samples (*n* = 4 mice) show spatial separation from sham treated samples (*n* = 4 samples) along PC1 employing principal component analysis (PCA). **b** Top 25 differentially expressed genes (DEG) between the indicated groups, scale bar indicates log2-fold changes of genes (*Z*-Scores; positive lgFC indicates higher gene expression in the respective group). **c** Gene set enrichment analysis (GSEA) indicates negative correlation to previously described tuft-1 (neuronal) and tuft-2 (immune) phenotypes following scopolamine treatment; ES enrichment score. **d** Instead, tuft cells strongly enrich for enteroendocrine cell lineage signatures. **e** GSEA analysis of published enteroendocrine signature gene sets shows the adoption of an enteroendocrine progenitor subtype as well as increased differentiation into early and late EC cells, L cells and N cells of expanding tuft cells^13,32^ (see Supplementary Data 1 for leading edge gene signatures); the enrichment for enterocyte signatures further confirms increased differentiation. EEC enteroendocrine cell, EC enterochromaffin cell, NES normalized enrichment score. **f** Master regulator analysis shows significant changes in transcription factor activity after scopolamine treatment, such as activation of *Srebf2* and inactivation of *Nme2*, respectively (*Z*-Scores; Srebf2 *p* = 0.00086; Hnf4a *p* = 0.0014; Ewsr1 *p* = 0.0019; Taf9 *p* = 0.0019; Nme2 *p* = 0.0015; Sarnp *p* = 0.0011; Scand1 *p* = 0.00012). FDR false discovery rate, Act differential activity (red indicating increased, blue indicating decreased), Exp differential gene expression.

ZSgreen-positive tuft cells from sham- and scopolamine-treated mice were strongly enriched for tuft cell markers, such as *Trpm5*, *Pou2f3* or *Dclk1* (Supplementary Fig. 4B). While the analysis detected only 165 differentially expressed genes (DEG) between the groups (Supplementary Fig. 4C), principal component analysis showed spatial separation of the samples by scopolamine treatment along PC1 (Fig. 3a), suggesting the induction of a distinct tuft phenotype. Indeed, analysis of the top DEG induced by scopolamine treatment showed an enrichment of genes important in neuroendocrine cell function (*Nkx6–3*, *Qpct*, *Abca2*, *Lmx1a*, *Nr4a1*) as well as synaptic activity (*Arc*, *Prss12*) (Fig. 3b). To our surprise, gene set enrichment analysis (GSEA) showed that scopolamine-treated tuft cells did not adopt a neuronal (tuft-1) or immune (tuft-2) phenotype as previously defined by Haber et al.^13^ (Fig. 3c), but instead adopted an enteroendocrine phenotype (Fig. 3d).

While substantial heterogeneity has been ascribed to intestinal tuft cells^11^, an enteroendocrine phenotype has not yet been described. However, these results are in line with the prominent tuft cell expansion observed in Prox1-CreERT2 × M3R fl/fl mice (Fig. 2e), given the known relationship of Prox1-positive cells to the enteroendocrine lineages^22^. Also, the negligible expression of muscarinic receptors in ZSgreen-positive tuft cells accords with previous data sets^13^ (Supplementary Fig. 4D) and with the observation that tuft cells are likely not the primary sensor cells of muscarinic blockade (see Supplementary Fig. 3B). A total of 12–14 subtypes of intestinal endocrine cell types have been identified^13,32^, and we were indeed able to detect enriched gene signatures indicating the adoption of an early EEC progenitor subtype, simultaneous to signatures from potentially functionally relevant differentiated endocrine subtypes such as L cells, EC cells (late, early) or N cells^13,32^ in scopolamine-treated tuft cells (Fig. 3e and Supplementary Data 1).

Finally, previously identified tuft lineage determination markers, such as *Atoh1*^19^, *Sox4*^20^, or *Pou2f3*^14^, did not show differential expression between the groups (see “Methods” section for deposited data information). *Atoh1* has been closely linked to secretory cell differentiation in the intestine^33^, and thus its stable expression between the groups is in line with enteroendocrine tuft differentiation. The fact that we observed almost negligible changes to previously reported important regulators of EEC differentiation such as *Neurog3* or *Sox4*, however, does not rule out that these may have possibly been expressed transiently at an earlier timepoint after treatment onset than at the time of analysis^32^.

To gain further insights into putative functionally active regulatory networks in the expanding tuft cells, we performed master regulatory network analysis^34^ (see “Methods” section for details), and the MARINa plot confirmed the significant activation of *Srebf2*, *Hnf4a*, and *Ewsr1* (Fig. 3f). While *Srebf2* appears mainly important for cellular cholesterol synthesis and transport^35^, cholesterol has been identified as an important regulator of enteroendocrine cell differentiation^36^ and driver of ISC proliferation^37^. Similarly, *Hnf4a* appears important for normal enterocyte and enteroendocrine cell maturation, and its genetic ablation in murine intestinal epithelium severely perturbs intestinal homeostasis^38^. With targets of *Ewsr1* being enriched in Bmi1-eGFP-positive endocrine cells^39^, this analysis confirmed the likelihood that expanding enteroendocrine tuft cells become functionally active in order to compensate for epithelial muscarinic receptor perturbations.

### Expanding tuft cells contribute to increased Ach secretion

**Fig. 4:**
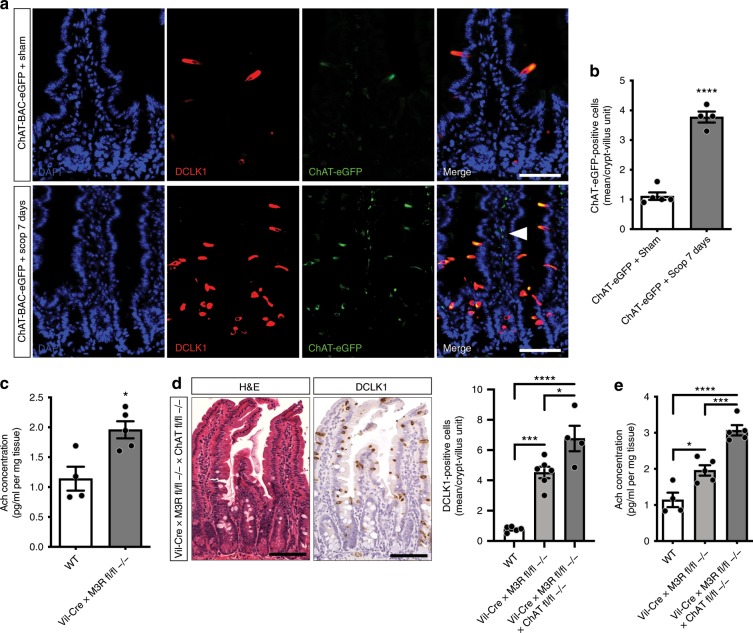
Expanding tuft cells orchestrate increased secretion of acetylcholine. **a**, **b** Scopolamine-treated (7 days) ChAT-BAC-eGFP mice showed expansion of ChAT-eGFP-/DCLK1-positive cells (*n* = 5 sham mice, Mean = 1.11, SEM = 0.125; *n* = 4 ChAT-BAC-eGFP scopolamine-treated mice, Mean = 3.775, SEM = 0.184; unpaired *t* test, two-tailed, *t* = 12.39, df = 7), and prominent cholinergic fibers (arrowhead); bar graphs = 50 µm. **c** Acetylcholine increases following epithelial M3R ablation (ELISA, whole small intestinal tissues; *n* = 4 WT mice, Mean = 1.14, SEM = 0.2; *n* = 5 Vil-Cre × M3R fl/fl −/− mice, Mean = 1.958, SEM = 0.143; unpaired *t* test, two-tailed, *t* = 3.425, df = 7). **d** Immunostainings of Vil-Cre × M3R fl/fl × ChAT fl/fl mice for DCLK1 (*n* = 5 WT mice, Mean = 0.77, SEM = 0.080; *n* = 6 Vil-Cre × M3R fl/fl −/− mice, Mean = 4.517, SEM = 0.377; *n* = 4 Vil-Cre × M3R fl/fl −/− × ChAT fl/fl −/− mice, Mean = 6.763, SEM = 0.844; ordinary one-way ANOVA, *F* = 38.95, df (total) = 14), bar graphs = 100 µm. **e** Acetylcholine increases in Vil-Cre × M3R fl/fl × ChAT fl/fl mice (*n* = 4 WT mice, Mean = 1.14, SEM = 0.2; *n* = 5 Vil-Cre × M3R fl/fl −/− mice, Mean = 1.958, SEM = 0.143; *n* = 5 Vil-Cre × M3R fl/fl −/− × ChAT fl/fl −/− mice, Mean = 3.07, SEM = 0.142; ordinary one-way ANOVA, *F* = 36.12, df (total) = 13). Source data are provided as a Source Data file. *p < 0.05, ***p < 0.005, ****p < 0.001.

Next, we aimed to address the functional relevance of the observed tuft cell expansion following muscarinic receptor blockade. Choline acetyltransferase (the gene encoding *Chat*) is the rate-limiting enzyme for the production of Ach, which is the main neurotransmitter of cholinergic neurons^3^ and modulates epithelial proliferation^7^. Within the intestinal epithelium, *Chat* expression is unique to tuft cells^10^, which was confirmed analyzing ZSgreen-positive vs. ZSgreen-negative epithelial cells by RT-PCR for *Chat* (Supplementary Fig. 4E). Employing scopolamine treatment in ChAT-BAC-eGFP mice showed strong co-labeling of ChAT-eGFP with DCLK1 (Fig. 4a), and ChAT-eGFP-positive cells expanded significantly (Fig. 4b) simultaneous to the more prominent appearance of ChAT-eGFP-positive stromal neuronal fibers following scopolamine treatment (Fig. 4a, white arrowhead).

Together, along with the observed enteroendocrine differentiation, this data suggested that the expanding tuft cells may orchestrate a compensatory circuit to increase cholinergic tone following muscarinic receptor blockade. Strikingly, Ach levels increased significantly within the intestinal mucosa of Vil-Cre × M3R fl/fl mice (Fig. 4c). The additional epithelial ablation of M1R did not result in increased Ach secretion (Supplementary Fig. 4F). To further assess the functional importance of the epithelial expression of *ChAT* in expanding tuft cells, we analyzed tissues from Vil-Cre × M3R fl/fl × ChAT fl/fl mice, which showed an even more pronounced expansion of DCLK1-positive tuft cells compared with Vil-Cre × M3R fl/fl alone (Fig. 4d), along with a dramatic increase in secreted Ach (Fig. 4e). This indicated increased secretion of Ach from the surrounding stroma possibly orchestrated by expanding tuft cells, which is supported by the increased presence of neuronal Beta-III-Tubulin-positive fibers in these samples (Supplementary Fig. 4G, arrows). In line with this hypothesis are data from organoids from Vil-Cre × M3R fl/fl compared with WT mice, which did not show expanded DCLK1-positive tuft cells, possibly indicating the importance of the epithelial-stromal crosstalk in this compensatory mechanism or an altered cholinergic niche dependency of cultured epithelial cells (Supplementary Fig. 4H).

### Increased Ach secretion sustains compensatory PI3K signaling

**Fig. 5:**
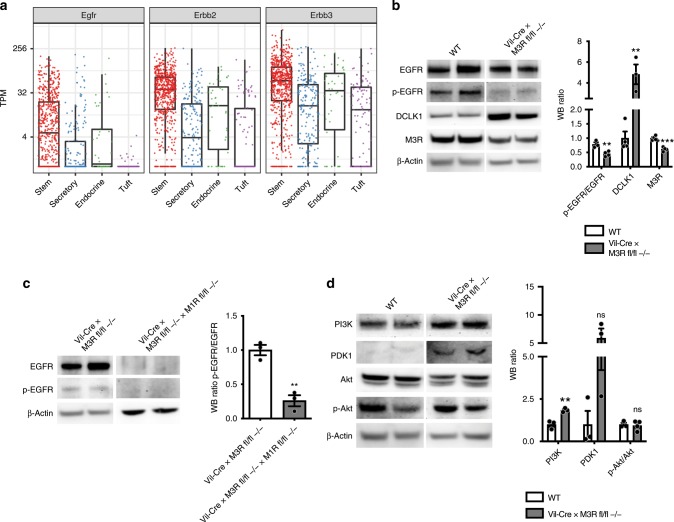
Cholinergic niche sustains compensatory PI3K signaling following epithelial cholinergic receptor ablation. **a** *EGFR*, *Erbb2*, and *Erbb3* expression in intestinal single cells^13^; TPM transcripts per kilobase million. **b** Immunoblot analyses of epithelial-enriched Vil-Cre × M3R fl/fl mouse samples for p-EGFR/EGFR, DCLK1, and M3R (*n* = 3 WT mice p-EGFR/EGFR; 4 WT mice DCLK1, M3R; *n* = 4 Vil-Cre × M3R fl/fl −/− p-EGFR/EGFR, M3R; 3 Vil-Cre × M3R fl/fl −/− mice DCLK1; WT p-EGFR/EGFR Mean = 0.783, SEM = 0.073; DCLK1 Mean = 1, SEM = 0.234; M3R Mean = 1, SEM = 0.04; Vil-Cre × M3R fl/fl p-EGFR/EGFR Mean = 0.45, SEM = 0.043; DCLK1 Mean = 4.827, SEM = 0.95; M3R Mean = 0.612, SEM = 0.036; multiple *t* tests; p-EGFR/EGFR *t* = 4.175, df = 5; DCLK1 *t* = 4.547, df = 5; M3R *t* = 7.177, df = 6). **c** Analysis of Vil-Cre × M3R fl/fl × M1R fl/fl mice shows additive downregulation of EGFR activation compared with Vil-Cre × M3R fl/fl mice (*n* = 3 Vil-Cre × M3R fl/fl −/− mice, *n* = 3 Vil-Cre × M3R fl/fl −/− × M1R fl/fl −/− mice; Vil-Cre × M3R fl/fl −/− p-EGFR/EGFR Mean = 1, SEM = 0.076; Vil-Cre × M3R fl/fl −/− × M1R fl/fl −/− p-EGFR/EGFR Mean = 0.260, SEM = 0.080; unpaired *t* test, two-tailed, *t* = 6.721, df = 4). **d** Epithelial M3R ablation sustained PI3K-PDK1 signaling (*n* = 3 WT, 3 Vil-Cre × M3R fl/fl −/− mice PI3K, PDK1; *n* = 3 WT mice p-Akt/Akt, *n* = 4 Vil-Cre × M3R fl/fl −/− mice p-Akt/Akt; WT PI3K Mean = 1, SEM = 0.144; PDK1 Mean = 1, SEM = 0.799; p-Akt/Akt Mean = 1, SEM = 0.103; Vil-Cre × M3R fl/fl −/− PI3K Mean = 1.844, SEM = 0.069; PDK1 Mean = 5.906, SEM = 1.697; p-Akt/Akt Mean = 0.941, SEM = 0.142; multiple *t* tests; PI3K *t* = 5.278, df = 4; PDK1 *t* = 2.617, df = 4, *p* = 0.059; p-Akt/Akt *t* = 0.3102, df = 5). Source data are provided as a Source Data file. *p < 0,05, **p < 0.01, ***p < 0,005.

Next, we wanted to test our hypothesis that the prominent increase of Ach in these samples may serve to maintain important intracellular signaling, as previous in vitro studies suggested a paracrine action by epithelial-derived Ach on ISC or progenitor cells^40^. GPCRs, such as M3R, are able to *trans*-activate EGFR^9^, and previous data sets^13^ showed prominent expression of EGF receptors in Lgr5-positive ISCs (Fig. 5a, stem compartment). Strikingly, immunoblot analysis of epithelial-enriched intestinal samples from Vil-Cre × M3R fl/fl mice showed a significant downregulation of p-EGFR, indicating decreased EGFR activation, next to an increase in DCLK1 and a decrease of M3R (Fig. 5b). These findings are highly reminiscent of the recent reports of Lgr5-positive ISC quiescence following EGF pathway inhibition in vitro, which was also associated with a selective expansion of DCLK1-positive tuft cells^41^. In line with the strong histologic phenotype (Supplementary Fig. 2E), analysis of Vil-Cre × M3R fl/fl × M1R fl/fl tissues showed a significant reduction of EGFR activation compared with Vil-Cre × M3R fl/fl alone (Fig. 5c).

In addition, we found that the nonselective cholinergic muscarinic receptor agonist carbachol partially rescued organoid survival following EGF withdrawal in vitro (Supplementary Fig. 5A), supporting the importance of muscarinic agonism for ISC growth and survival. Despite the strong deactivation of EGFR, intracellular levels of ERK/p-ERK or TCF-1/7 did not significantly change in Vil-Cre × M3R fl/fl tissues (Supplementary Fig. 5B), thus indicating that increased Ach levels may maintain intracellular signaling pathways.

Facilitation of neuronal cell differentiation from pluripotent progenitor cells has been associated with upregulation of PI3K/Akt/mTOR signaling^42^, and indeed we could find the genes *Pi3kcd* and *Tns2* among the top DEG in our sequencing analysis (Fig. 3b), suggesting the activation of PI3K signaling following scopolamine treatment. Indeed, immunoblot analyses of Vil-Cre × M3R fl/fl mice showed an upregulation of the PI3K-PDK1 axis, while activation of Akt remained unchanged (Fig. 5d), thus indicating a possible compensatory shift in intracellular signaling activation following cholinergic receptor blockade and reduced EGFR activation.

### Acute tissue injury abrogates compensatory tuft cell circuit

**Fig. 6:**
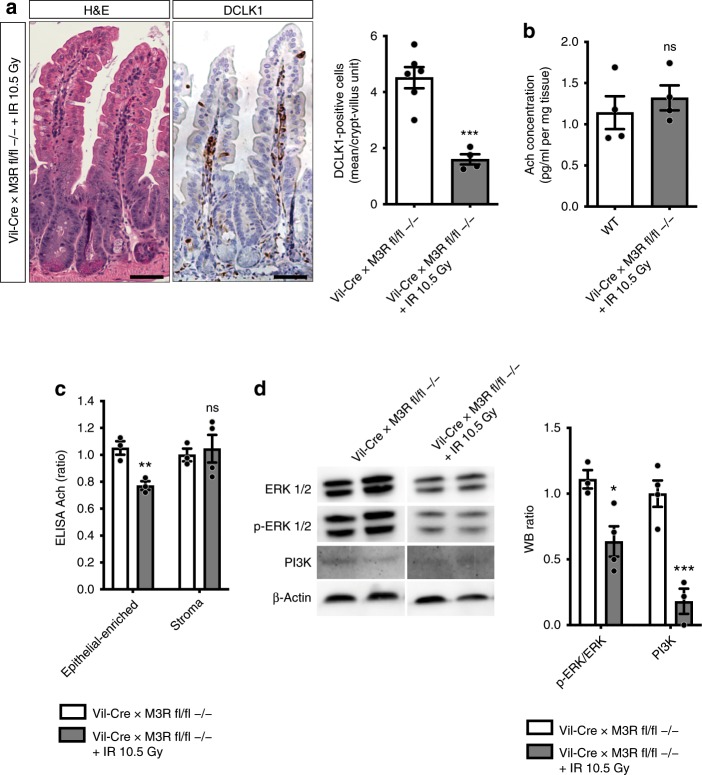
Acute tissue injury abrogates compensatory tuft cell circuit following epithelial M3R ablation. **a** H&E and DCLK1 immunostainings 3 days after 10.5 Gy WBI (*n* = 6 Vil-Cre × M3R fl/fl −/− mice, Mean = 4.517, SEM = 0.377; *n* = 4 Vil-Cre × M3R fl/fl −/− + IR 10.5 Gy mice, Mean = 1.6, SEM = 0.185; unpaired two-tailed *t* test, *t* = 5.906, df = 8), bar graphs = 100 µm. **b** Acetylcholine in Vil-Cre × M3R fl/fl tissues 3 days after 10.5 Gy WBI (ELISA; *n* = 4 mice/group; WT Mean = 1.14, SEM = 0.2; Vil-Cre × M3R fl/fl −/− + IR 10.5 Gy Mean = 1.321, 0.152; unpaired *t* test, *t* = 0.7189, df = 6). **c** ELISA of epithelial-enriched vs. stroma-enriched Vil-Cre × M3R fl/fl tissues 3 days after 10.5 Gy WBI (*n* = 3 Vil-Cre × M3R fl/fl −/− controls epithelial-enriched, stroma; *n* = 3 Vil-Cre × M3R fl/fl −/− + IR epithelial-enriched, *n* = 4 Vil-Cre × M3R fl/fl −/− + IR stroma; Vil-Cre × M3R fl/fl −/− control epithelial-enriched Mean = 1.051, SEM = 0.05; stroma Mean = 1, SEM = 0.047; Vil-Cre × M3R fl/fl −/− + IR 10.5 Gy epithelial-enriched Mean = 0.772, SEM = 0.032; stroma Mean = 1.046, SEM = 0.102; multiple *t* tests, epithelial-enriched *t* = 4.706, df = 4; stroma *t* = 0.3609, df = 5). **d** p-ERK/ERK and PI3K analysis after acute injury (3 days post 10.5 Gy WBI; *n* = 3 Vil-Cre × M3R fl/fl −/− controls p-ERK/ERK, *n* = 4 Vil-Cre × M3R fl/fl −/− controls PI3K; *n* = 4 Vil-Cre × M3R fl/fl −/− + IR p-ERK/ERK, *n* = 3 Vil-Cre × M3R fl/fl −/− + IR PI3K; Vil-Cre × M3R fl/fl −/− control p-ERK/ERK Mean = 1.109, SEM = 0.07; PI3K Mean = 1, SEM = 0.101; Vil-Cre × M3R fl/fl −/− + IR p-ERK/ERK Mean = 0.637, SEM = 0.115; PI3K Mean = 0.181, SEM = 0.096; ordinary two-way ANOVA, p-ERK/ERK *t* = 3.236, df = 10; PI3K *t* = 5.608, df = 10). Source data are provided as a Source Data file. *p < 0.05, **p < 0.01, ***p < 0.005; ns not significant.

While a marked expansion of enteroendocrine tuft cells with the accompanying increase in Ach secretion can likely maintain homeostasis following moderate reductions in muscarinic receptor expression and cholinergic signaling, this compensatory mechanism appears to rapidly collapse following more severe acute tissue injury. When Vil-Cre × M3R fl/fl mice were subjected to acute whole body irradiation of 10.5 Gy, we observed a dramatic loss of epithelial tuft cells concomitant to severely altered intestinal tissue architecture in these mice (Fig. 6a). This also coincided with a striking decrease in Ach secretion, which was reduced to WT levels (Fig. 6b). As cholinergic neurons have been found to suppress Ach secretion in response to acute intestinal inflammation^43^, we next aimed to address the contribution of either the epithelial or stromal compartment in these mice to the observed decrease in Ach. Strikingly, we detected an isolated, marked decrease of Ach in the epithelial compartment of irradiated Vil-Cre × M3R fl/fl mice, with no changes to stromal Ach (Fig. 6c).

Pretreatment of these mice with the broad cholinergic receptor agonist bethanechol did only moderately reduce DCLK1-positive tuft cell number, but dramatically improved tissue morphology (Supplementary Fig. 5C), supporting the importance of Ach for intestinal homeostasis. Finally, with the loss of the compensatory tuft expansion and mucosal Ach secretion following irradiation in Vil-Cre × M3R fl/fl mice, we observed a striking decrease in intestinal epithelial intracellular EGF and PI3K pathway activity (Fig. 6d). Collectively, these findings strongly support the importance of epithelial tuft cells as epithelial niche cells, and are consistent with previous observations regarding the importance of M3R signaling for intestinal homeostasis^24^.

## Discussion

Here, we have shown the importance of DCLK1-positive tuft cells in orchestrating a cholinergic niche, providing Ach to Lgr5-positive ISCs as well as secretory cells to maintain epithelial homeostasis. Prox1-positive cells appear as the key cholinergic sensing cells, as they are able to direct the expansion of an enteroendocrine tuft phenotype upon interruption of cholinergic signal. While Prox1-positive cells appear as the most probable sensor cells, it is important to note that subpopulations of tuft cells have also been reported to express *Chrm3* and *Prox1*^13^. Even if these transcripts were only transiently expressed by tuft cell subpopulations, this could enable tuft cells to also sense cholinergic signaling modulation. Yet, since lineage tracings did not show the expansion of traced tuft cells in response to muscarinic receptor blockade (Supplementary Fig. 3B), tuft cell sensing similar to Prox1-positive cell sensing would require their signaling to yet another cell of origin for the expanding enteroendocrine tuft cells.

Our RNA-sequencing analysis showed that the expanding tuft cells following scopolamine treatment express important genes for enteroendocrine cell function, in conjunction with hints at synaptic activity and signal transmission. It is important to note that the leading edge genes from scopolamine-treated tuft cells which overlap with previously defined endocrine cell signatures do not show altered expression of PYY or ChgA, thus possibly explaining the reason we did not observe an expansion of PYY- or ChgA-positive endocrine cell types in Vil-Cre × M3R fl/fl mice (Supplementary Fig. 2B, C). Intestinal endocrine cells appear to communicate with neighboring afferent neuronal cells^44,45^, thus providing tuft cells with several possible avenues for increasing Ach secretion in response to tissue perturbations. In particular, we observed the robust expansion of ChAT-eGFP-/DCLK1-positive tuft cells following scopolamine treatment (see Fig. 4a, b). However, in the setting of more severe intestinal injury (g-irradiation) with loss of tuft cells, there is a prominent, epithelial-restricted loss of Ach (see Fig. 6c). Taken together, expanding tuft cells are ideally suited to produce and secrete Ach into the mucosal niche.

On the other hand, the adoption of an enteroendocrine signature may also indicate that expanding tuft cells orchestrate a stromal circuit, possibly involving mucosal neuronal cells to increase overall Ach release. Indeed, submucosal cholinergic nerve fibers have been reported to govern epithelial proliferation^7^. This is supported by the emergence of strong ChAT-eGFP-positive stromal fibers following scopolamine treatment (Fig. 4a). Furthermore, the observation that genetic ablation of ChAT in Vil-Cre × M3R fl/fl mice results in a strong increase of secreted Ach suggests that functional tuft cells in the M3R deficient mice are indeed able to compensate for the loss of muscarinic signaling by secreting Ach. However, upon loss of epithelial ChAT and thus epithelial Ach production, stromal cells necessarily upregulate Ach secretion, possibly in response to signaling from expanding tuft cells in these mice (Fig. 4d, e).

Whether or not immune, neuronal or enteroendocrine tuft phenotypes are entirely functionally distinct remains to be clarified. Indeed, Ach is considered one of the major transmitters mediating neuroimmune crosstalk^46^. The diminished type 2 immune response to Helminth infection reported in whole body M3R-KO^24^ may thus also originate from impaired Ach signaling on innate lymphoid cells^47^ and decreased IL-13 release, which appears as the main trigger of goblet and tuft cell hyperplasia^15^. Consequently, if helminth infection of mice bearing an epithelial-specific genetic M3R ablation yielded similar results to M3R-KO mice, cholinergic modulations would indeed direct an overriding enteroendocrine tuft cell expansion.

**Fig. 7:**
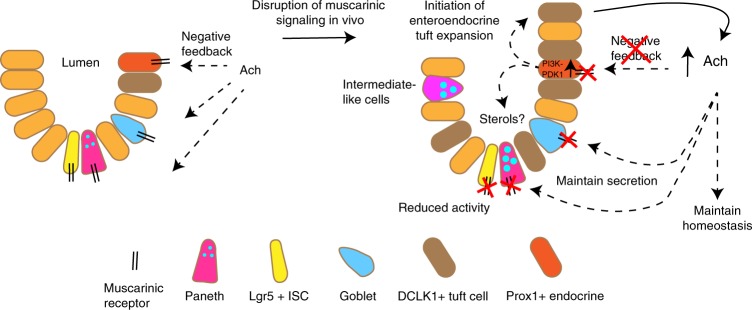
Model summarizing the importance of the cholinergic intestinal niche to maintain epithelial homeostasis. Prox1-positive endocrine cells respond to a loss of negative feedback upon M3R ablation, which results in the expansion of an enteroendocrine tuft cell phenotype and sustained cholinergic niche signaling.

The primary sensing of epithelial cholinergic interruption appears to take place in lineage-committed cells, while its effect on Lgr5-positive ISC rather resembles previously identified roles of GPCRs in regulating ISC kinetics. Pharmacologic modifications of intestinal Lgr5-positive ISC in Rspo gain-of-function models demonstrated the induction of Lgr5-positive ISC self-renewal, while Rspo loss-of-function initiated a default lineage commitment^48^. Furthermore, peptide inhibition of Fzd7 has been shown to effectively decrease ISC activity and Lgr5-EGFP-positive ISC count in vitro^49^. The latter in particular is reminiscent of the effect of pharmacologic muscarinic receptor blockade we observed on Lgr5-EGFP-positive cells, which were significantly reduced in vivo with only short-term treatment. Thus, our data uncover an additional epithelial compensatory circuit centering around tuft cells in support of their emerging importance as epithelial niche cells^31^ (Fig. 7). Furthermore, our findings add to the importance of GPCRs and the potential of GPCR-targeted therapy to alter ISC behavior in homeostasis and disease.

## Methods

### Animal models

Adult mice were aged between 6 and 10 weeks at the time of sacrifice or tamoxifen induction, both sexes were used. C57BL/6J WT (stock nr. 00064), Vil-Cre (stock nr. 021504), ChAT-BAC-eGFP (stock nr. 007902), Prox1-CreERT2 (stock nr. 022075), Lgr5-EGFP-IRES-CreERT2 (stock nr. 008875), ChAT-loxp-stop-loxp (ChAT fl/fl; stock nr. 016920), Rosa26-CAG-loxp-stop-loxp-tdTomato (tdTom; stock nr. 007909), and Rosa26-loxp-stop-loxp-mTmG (TGFP; stock nr. 007676) were all obtained from the Jackson Laboratory (USA). M3R-KO^50^ were procured from Duan Chen (Norway), M1R-KO and M3-loxp-stop-loxp (M3R fl/fl) mice were a kind gift from J. Wess (NIH, USA), Lgr5-EGFP-DTR mice were procured from Genentech (USA) and M1-loxp-stop-loxp (M1R fl/fl) mice were a kind gift from S. Tonegawa (RIKEN-MIT, USA). Constitutive or conditional genetic ablation of M3R and/or M1R and/or ChAT in the indicated mouse models did not alter viability, health, behavior, or reproduction of experimental mice. Dclk1-BAC-CreERT mice were generated in our lab^18^. To generate Dclk1-DTR-ZsGreen reporter mice, the *DTR-2A-Zsgreen-pA-FrtNeoFrt* cassette was ligated into a pL451 plasmid and subsequently electroporated into the ATG-containing exon 2 of the mouse *Dclk1* gene on SW105 *Dclk1-*BAC-carrying cells (clone RP23-283D6). Isolated and linearized BAC DNA was subsequently microinjected into the pronucleus of fertilized CBA × C57BL/6J oocytes at the Columbia University Transgenic Animal Core facility, with subsequent backcrossing to C57BL/6J mice (full characterization of the mice will be reported elsewhere). All analyses have been performed in the jejunum since previous studies identified the presence of distinct stem cell pools in this region^51–54^ and in order to reduce variability and increase reproducibility between studies^55^. Mice were housed in a specific pathogen-free facility. All mouse studies were approved by the Columbia University Institutional Animal Care and Use Committee (directed by Mary Jo Shepherd, Execute Director of the Office of the IACUC, Columbia University).

### Mouse treatments

Treatment with the nonselective cholinergic muscarinic receptor antagonist scopolamine (Santa Cruz) was performed using implantation of osmotic pumps (Alzet pump models 2001 and 2006, DURECT Corporation). Briefly, mice aged 8–10 weeks were anesthetized using isoflurane and pumps were implanted into the flank of experimental mice. The treatment dose was 0.5 mg/KG BW/day^7^, sodium chloride was used for sham controls. Postoperative anesthesia and monitoring was provided according to Columbia University animal protocol number AC-AAAZ7450. To induce reporter recombination in susceptible mouse lines, mice were induced once with 3 mg tamoxifen (Sigma) dissolved into corn oil by oral gavage. Bethanechol (Sigma-Aldrich) was administered to experimental mice at a dose of 400 µg/ml in drinking water for the duration of 7 days^56^. Whole body acute irradiation injury (10.5 Gy) has been performed on a Mark I Cesium-137-based gamma-ray irradiator (J.L. Shepherd & Associates, San Fernando, USA) and postinterventional monitoring was performed according to Columbia University animal protocols (AC-AAAV8500, AC-AAAV1452, AC-AAAR3405).

### RT-PCR

Whole tissue preparations or >100,000 sorted cells were processed with the Macherey Nagel Nucleospin RNA extraction kit following the manufacturer’s protocol. Small sorted epithelial cell populations (<10,000 cells) were collected in lysis buffer and processed using the RNeasy Plus Micro kit (Qiagen) following the manufacturer’s protocol. RNA concentrations were measured using NanoDrop 2000 (Thermo Fisher Scientific). cDNA was synthesized with random hexamers using SuperScript III Reverse Transcriptase (Life Technologies). Gene expression was determined using PowerUp SYBR Green master mix (Applied Biosystems). Primers for measuring cDNA expression are listed in Supplementary Table 1. Quantitative PCR (RT-PCR) was performed on an Applied Biosystems QuantStudio 3 machine. Relative gene expression was normalized to either *Hmbs* or *Actb*.

### In vitro cultures

Small intestinal crypts were isolated from proximal murine jejunum^13^. Harvested pieces were minced, washed in ice-cold DPBS, subjected to EDTA chelation (10 mM solution, exchanged three times), and mild shaking at 4 °C. The suspensions were then filtered (70 µm), pelleted and resuspended in matrigel (Corning). Droplets of 25 µl were plated on 48-well plates (Corning) and overlayed with complete growth media (Advanced DMEM/F12 Invitrogen) containing hepes, glutamax, anti-anti, b-27, n-2 (all Invitrogen), 1 µM n-acetylcysteine (Sigma-Aldrich), 50 ng/mL EGF (Invitrogen), 100 ng/mL noggin (Peprotech), and 100 ng/mL R-spondin1 (gift from Chandan Guha, Einstein College of Medicine, New York). For immunofluorescence imaging, organoids were plated on eight-well chamber slides^57^. Carbachol (Sigma-Aldrich) was added to the media as indicated. Media were replaced every day and imaging was performed at the indicated timepoints.

### Flow cytometry

Small intestinal crypts and villi were processed as described above, with an additional step of mild pipetting using DPBS/5% FBS solution following EDTA chelation to increase crypt cell yield. Single-cell suspensions were then obtained from enriched crypts and villi by incubation in culture media containing ROCK inhibitor for 45 min at 37 °C, followed by mild mechanical dissociation using a syringe with a 21G needle^58^. The suspension was then filtered through a 40 µm mesh and dissociation and cell count determined using a hemocytometer. Stainings were performed with fluorophore-conjugated antibodies in 2% FBS/PBS solution for 25 min on ice in the dark (see Supplementary Table 2 for used antibodies). Live/dead staining has been achieved by addition of DAPI right before data acquisition on a BD Fortessa flow cytometer. Flow cytometry assisted cell sorting (FACS) has been performed using FACS Aria II. FACS Diva Software and FlowJo V10 has been employed to process and analyze data.

### Immunohistochemistry

For immunohistochemistry, slides were deparaffinized in xylene and dehydrated in ethanol. Antigen retrieval was performed by boiling the slides in citrate buffer (10 mM, pH 6.0; Vector laboratories) in a microwave for 15 min. Endogenous peroxidase was blocked by incubation with 3% hydrogen peroxide (Sigma-Aldrich). Slides were rinsed in 1% PBS/TritonX-100 (Fisher Scientific) and blocked for 30 min with 2% BSA (Fisher Scientific) and 10% second antibody serum. Primary antibodies were diluted in 2% BSA and incubated overnight at 4 °C. Biotinylated secondary antibodies (Vector laboratories) were incubated for 30 min at RT. Subsequently, slides were incubated with the ABC kit (Vector laboratories) and visualized using 3,3′-diaminobenzidine (Sigma-Aldrich) as chromogen. Slides were counterstained with hematoxylin, rehydrated in ethanol and mounted for viewing. Bright-field images were acquired using an Eclipse TU2000-U microscope (Nikon) connected to a cooled color CCD camera (Diagnostic Instruments) using SPOT software. Employed antibodies are listed in Supplementary Table 2, alcian blue staining has been achieved using the Abcam mucin stain kit (ab150662). For quantifications, positively stained cells in jejunal tissues were counted in at least 10–20 crypt–villus units per mouse.

### Immunofluorescence

Dissected mouse intestinal tissues were fixed in 4% PFA, embedded in OCT, and snap frozen in liquid nitrogen. Washed slides were permeabilized and blocked simultaneously with 1% TritonX-100/PBS/1% BSA for 1 h at RT. Primary antibodies were applied for overnight staining at 4 °C in 1% BSA/PBS. Alexa Fluor secondary antibodies (Invitrogen) at a concentration of 1:500 were used to reveal staining. Slides were counterstained and mounted with Vectashield anti-fade DAPI-containing mounting medium (Vector Laboratories). Fluorescence images were acquired using an Eclipse TU2000-U microscope (Nikon). Organoid staining has been achieved following fixation in 4% PFA at RT, wash with PBS and subsequent simultaneous permeabilization, and blocking with 0.5% TritonX-100/PBS/5% BSA for 1 h at RT^59^. Primary antibody staining was achieved overnight at 4 °C in 1% BSA/PBS. Alexa Fluor secondary antibodies (Invitrogen) at a concentration of 1:500 were used to reveal staining. Fluorescence images were acquired using a Spinning disk confocal microscope (Zeiss). Employed antibodies are listed in Supplementary Table 2.

### Sample preparation for bulk RNA-sequencing analysis

Dclk1-DTR-ZSgreen mice have been treated with scopolamine or sodium chloride (Sham) for 7 days and sacrificed for tissue collection and subsequent cell isolation. Live/EPC-high ZSgreen-positive cells have been sorted on a FACS Aria II sorter and mRNA was processed employing the RNeasy Plus Micro kit (Qiagen). Sample quality (RIN) has been measured using Agilent Bioanalyzer and all analyzed and sequenced samples had a RIN of 9 or above. The Clontech Ultra Low v4 kit was used for cDNA amplification using 200–1000 pg of total RNA as input and 10–15 cycles amplification. 150 pg of cDNA was then prepared for sequencing using the Nextera XT protocol according to the manufacturer’s instructions. Libraries were sequenced to a depth of 40 M 100 bp paired end reads on the Illumina NovaSeq 6000 at Columbia Genome Center.

### Bulk RNA-sequencing gene expression and GSEA analysis

RTA (Illumina) was used for base calling and bcl2fastq2 (version 2.20) for converting BCL to fastq format, coupled with adaptor trimming. Pseudoalignment was carried out to a kallisto index created from the murine GRCm38 transcriptome using kallisto (0.44.0). Estimated counts and transcripts per kilobase million (TPM) per gene were computed from the kallisto output using the *tximport* R package^60^. To test for DEG between Sham and Scopolamine-treated tuft cells a negative binomial generalized linear model was used as implemented in the *DESeq2* R package^61^ and a false discovery rate < 0.1 was considered significant. The expression in TPM for select genes was illustrated in a heatmap using the *pheatmap* R package (https://CRAN.R-project.org/package=pheatmap). Consensus signature genes for subsets of epithelial cells were retrieved from the supplement of Haber et al.^13^ and Gehart et al.^32^ and their enrichment in the gene expression signature between Scopolamine and Sham treated tuft cells was examined using methodology as described by Subramanian et al.^62^.

### Tuft cell regulatory model and master regulator analysis

A tuft cell regulatory network was reverse engineered by ARACNe-AP^63^ using 102 single-cell RNA-Seq gene expression profiles of the full length dataset from Haber et al.^13^. Genes with less than one transcript per kilobase million in at least 10 of the 102 tuft cells were removed. ARACNe was run with standard settings (using data processing inequality (DPI), with 100 bootstrap iterations using all gene symbols mapping to a set of 1813 transcription factors that includes genes annotated in the Gene Ontology (GO) molecular function database as GO:0003700 (transcription factor activity), GO:0004677 (DNA binding), GO:0030528 (transcription regulator activity), or as GO:0004677/GO: 0045449 (regulation of transcription). Thresholds for the tolerated DPI and mutual information *p* value were set to 0 and 10–8, respectively. Master regulator analysis was performed by interrogating the gene expression signature between scopolamine and sham treated tuft cells using the tuft cell regulatory network and the msviper algorithm as implemented in the *viper* R package^34,64^.

### Analysis of murine gut single-cell gene expression

Single-cell gene expression from gut epithelium as described by Haber et al.^13^ were retrieved from the Broad Institute’s Single-Cell Portal and only the full length RNA-sequencing data were used to assess the expression (in TPM) and detection rate (defined as fraction of cells per cell type with TPM > 0 for a given gene) of muscarinic receptor genes (Chrm1–4, Chrm5 was not annotated/assessed in the dataset). Furthermore, we looked at single-cell gene expression from Prox1- and Bmi1-positive, as well as Lgr5-negative and Lgr5-positive gut cell populations as described by Yan et al.^22^, which were retrieved from the respective NCBI GEO repository. Data were processed using the Seurat R package^65^ keeping only cells with at least 500 and at most 5000 detected genes and at most 25% mitochondrial reads with subsequent log-normalization and a scaling factor of 1e6. Detection rates as described above were compared between the indicated single-cell populations for members of the Egfr family annotated in the data.

### ELISA

Ach ELISA (Cat.-nr. E4453-100, Biovision) was performed according to the manufacturer’s instruction. Briefly, small intestinal tissue was washed and homogenized in PBS. Samples were diluted 1:5 in dilution buffer and conjugated with HRP-conjugate. After 60 min of incubation on ice in the dark, wells were washed and absorbance was measured at 450 nm after applying chromogen solution. Protein concentration was calculated using a supplied standard curve.

### Western blot

Small intestine was harvested and epithelial-enriched isolates obtained using EDTA chelation and filtration as described above. The enriched epithelial tissue was resuspended in RIPA Buffer (Alfa Aesar) containing phosphatase and protease inhibitors (Sigma-Aldrich). Tissue was homogenized using magnetic beads and incubated at 4 °C under constant agitation followed by centrifugation at 12,000 × *g* for 20 min. The supernatant was collected and protein concentration was determined by the Bio-Rad Protein Assay. Protein samples were mixed with SDS sample buffer (Novex Invitrogen) and reducing agent (Novex NuPage) and heated to 95 °C for 5 min. For immunoblotting, 50 µg of protein were loaded on a 8–12% bis-tris gel (Invitrogen) and electrophoresis was carried out for 150 min at 80 V at 4 °C. Protein was transferred on a PVDF membrane (Invitrogen) at 40 V overnight at 4 °C and blocked with 5% skim milk (Difco). Primary antibodies were diluted in skim milk and membranes were incubated overnight at 4 °C (see Supplementary Table 3 for antibodies). Following incubation, membranes were washed with TBS-T and incubated with secondary antibody (ECL donkey anti-rabbit) for 1 h at RT. Blotted membranes were washed with TBS-T and developed by using pierce blotting substrate using a developing machine (FlurChem M, Protein Simple, San Jose, USA). Quantification of imaging was performed using ImageJ (https://imagej.nih.gov). Uncropped immunoblot images including molecular weight size markers can be found in the Source Data file.

### Statistical analysis

Analyses were performed using GraphPad Prism® Software. Statistical measures include mean values, standard error of the mean, unpaired *t* test calculations, multiple *t* test calculations, and one-/two-way ANOVA analyses (adjusted for Tukey’s or Sidak’s multiple comparisons, respectively). Statistical significance is defined for *p* values < 0.05 between groups.

### Reporting summary

Further information on research design is available in the Nature Research Reporting Summary linked to this article.

## Supplementary information

Supplementary Information

Supplementary Dataset 1

Description of Additional Supplementary Files

Reporting Summary

## Data Availability

The authors declare that all data supporting the findings of this study are available within the article and its supplementary information files or from the corresponding author upon reasonable request. The RNA-sequencing data reported in this study are available from Gene Expression Omnibus with accession code GSE138365. The source data underlying Figs. 1B–D, 2C–E, 3, 4B–E, 5B–D, 6A–D, Supplementary Figs. 1A, B, D, E, 2A–E, 3E, 4E, F, H, and 5A–C are provided as a Source Data file.

## Source data

Source Data file

## Code availability

Select enrichment results were illustrated using custom R code available under https://github.com/fossbert/binilib.
