# Supplementary material for: Prox1-positive cells monitor and sustain the murine intestinal epithelial cholinergic niche
Source: Nat Commun. 2020 Jan 8;11:111. doi: 10.1038/s41467-019-13850-7 (PMC6949263; doi:10.1038/s41467-019-13850-7)
Supplement: Supplementary file 1 — Supplementary Information [file 41467_2019_13850_MOESM1_ESM.pdf]

Supplementary Information

Prox1-positive cells monitor and sustain the murine intestinal epithelial cholinergic niche

Middelhoff et al.

Supplementary Materials:

Supplementary Figures 1-5 and figure legends

Supplementary Tables 1-3

Supplementary Data 1 (separate)

Source Data file (separate)

Supplementary References

Supplementary Figure 1

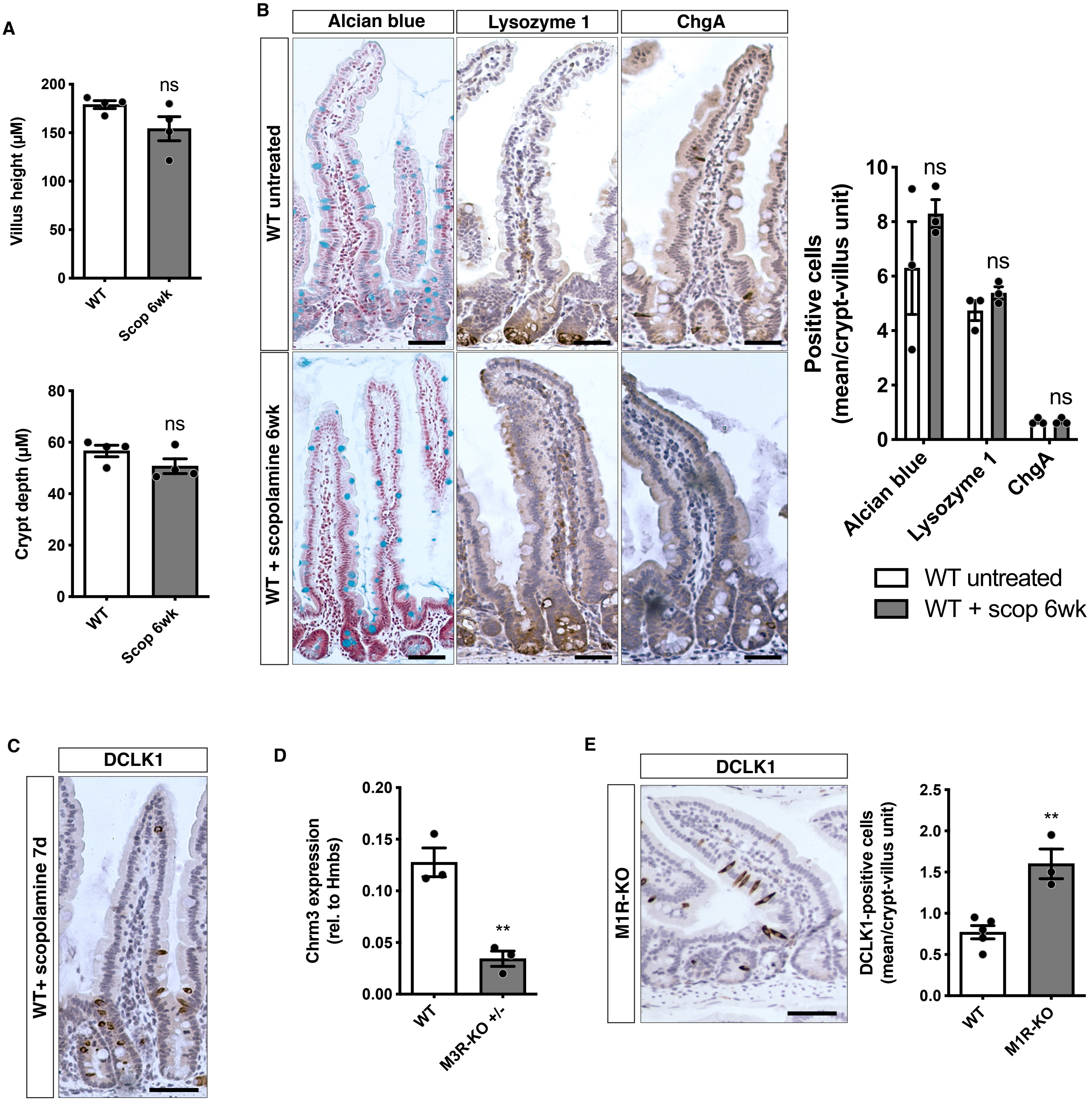

Supplementary Figure 1 related to Fig. 1. Muscarinic receptor blockade results in selective DCLK1-positive tuft cell expansion. (A) Long-term (6wk) scopolamine treatment of WT mice did not change intestinal tissue morphology in regards to villus height (n=4 mice each group; WT Mean = 178.997, SEM = 4.061; WT + scopolamine 6wks Mean = 154.15, SEM = 12.387; unpaired t test, two-tailed, t=1.906, df=6) or crypt depth (n=4 mice each group; WT Mean = 56.631, SEM = 2.232; WT + scopolamine 6wks Mean = 50.69, SEM = 2.849; unpaired t test, two-tailed, t=1.642, df=6). (B) Also, we could not observe significant changes to goblet cells (Alcian blue), Paneth cells (Lysozyme 1) or enterochromaffin cells (ChgA) following scopolamine treatment (n=3 WT mice for Alcian blue, Lysozyme 1, n=6 WT mice for ChgA; n=3 WT + scop 6wk mice for Alcian blue, n=4 WT + scop 6wk mice for Lysozyme 1, ChgA; WT Alcian blue Mean = 6.3, SEM = 1.704; Lysozyme 1 Mean = 4.733, SEM = 0.367; ChgA Mean = 0.608, SEM = 0.047; WT + scop 6wks Alcian blue Mean = 8.3, SEM = 0.513; Lysozyme 1 Mean = 5.175, SEM = 0.265; ChgA Mean = 0.663, SEM = 0.047; ordinary two-way ANOVA, Alcian blue t=2.217, df=17; Lysozyme 1 t=0.5235, df=17; ChgA t=0.07597, df=17), bar graphs = 100 µm. (C) DCLK1-positive tuft cell expansion also occurred following short-term scopolamine treatment (7d), bar graph = 50 µm. (D) RT-PCR analysis of jejunal tissues from heterozygous M3R-KO mice confirmed significant reduction of *Chrm3* expression (n=3 mice of each group; WT Mean = 0.128, SEM = 0.014; M3R-KO het Mean = 0.034, SEM = 0.007; unpaired t test, two-tailed, t=5.933, df=4). (E) Jejunal tissues of homozygous M1R-KO mice showed modest DCLK1-positive tuft cell expansion (n=5 WT mice, n=3 M1R-KO mice; WT Mean = 0.77, SEM = 0.080; M1R-KO Mean = 1.6, SEM = 0.18; unpaired t test, two-tailed, t=4.898, df=6), bar graph = 50 µm. Source data are provided as a Source Data file. ** = p < 0,01, ns = not significant.

Supplementary Figure 2

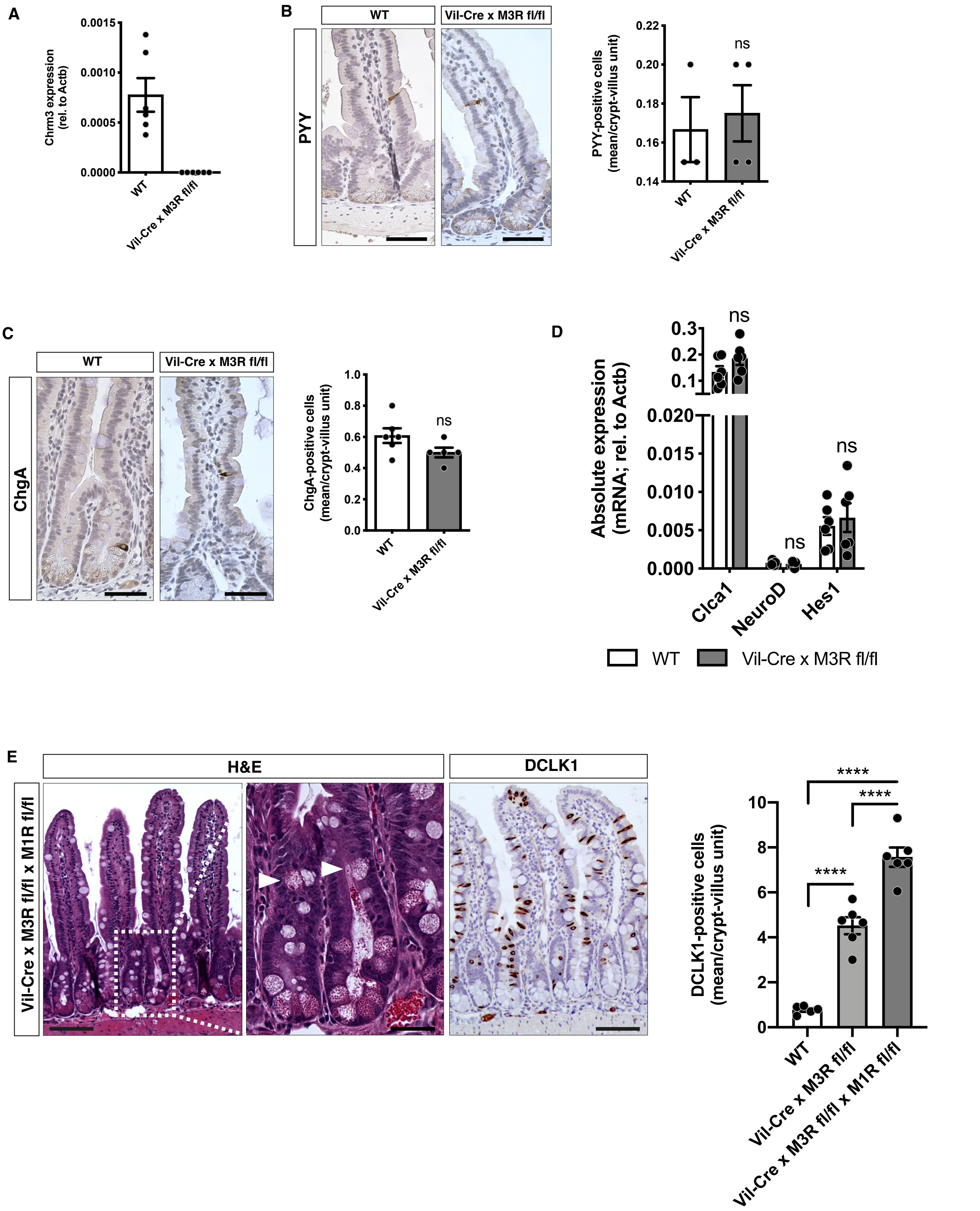

Supplementary Figure 2 related to Fig. 1. The epithelial compartment senses cholinergic interruption and initiates selective DCLK1-positive tuft cell expansion. (A) RT-PCR analyses of epithelial-enriched samples from Vil-Cre x M3R fl/fl mice for *Chrm3* transcript (n=6 WT and Vil-Cre x M3R fl/fl mice); n.d. = not detectable. (B, C) Immunohistochemical stainings for enteroendocrine (PYY; n=3 WT, n=4 Vil-Cre x M3R fl/fl mice; WT Mean = 0.167, SEM = 0.017; Vil-Cre x M3R fl/fl Mean = 0.175, SEM = 0.014; unpaired t test, two-tailed, t=0.3780, df=5) as well as enterochromaffin (ChgA; n=6 WT, n=5 Vil-Cre x M3R fl/fl mice; WT Mean = 0.608, SEM = 0.047; Vil-Cre x M3R fl/fl Mean = 0.5, SEM = 0.032; unpaired t test, two-tailed, t=1.189, df=9) cell subtypes following epithelial M3R ablation; bar graphs = 50 µm. (D) RT-PCR analyses of epithelial-enriched samples from Vil-Cre x M3R fl/fl mice for transcripts associated with secretory (*Clca1*), absorptive (*Hes1*) or endocrine cell (*NeuroD1*) development (n=6 WT and Vil-Cre x M3R fl/fl mice; WT Clca1 Mean = 0.133, SEM = 0.022; NeuroD Mean = 0.001, SEM = 0.0001; Hes1 Mean = 0.006, SEM = 0.001; Vil-Cre x M3R fl/fl Clca1 Mean = 0.186, SEM = 0.025; NeuroD Mean = 0.001, SEM = 0.0001; Hes1 Mean = 0.007, SEM = 0.002; multiple t tests, Clca1 t=1.565, df=10; NeuroD t=0.7157, df=10; Hes1 t=0.4921, df=10). (E) Vil-Cre x M3R fl/fl x M1R fl/fl mice showed a dramatic expansion of DCLK1-positive tuft cells compared to WT mice (n=5 WT mice, n=6 Vil-Cre x M3R fl/fl and Vil-Cre x M3R fl/fl x M1R fl/fl mice; WT Mean = 0.77, SEM = 0.080; Vil-Cre x M3R fl/fl Mean = 4.517, SEM = 0.377; Vil-Cre x M3R fl/fl x M1R fl/fl Mean = 7.567, SEM = 0.429; ordinary one-way ANOVA, F=88.82, df (total)=16); inset shows magnification of misplaced Paneth-like cells along the crypts reminiscent of intermediate cells (white arrowheads); bar graph left, right = 100 µm, bar graph center (magnification) = 25 µm. Source data are provided as a Source Data file. **** = p < 0,001, ns = not significant.

Supplementary Figure 3

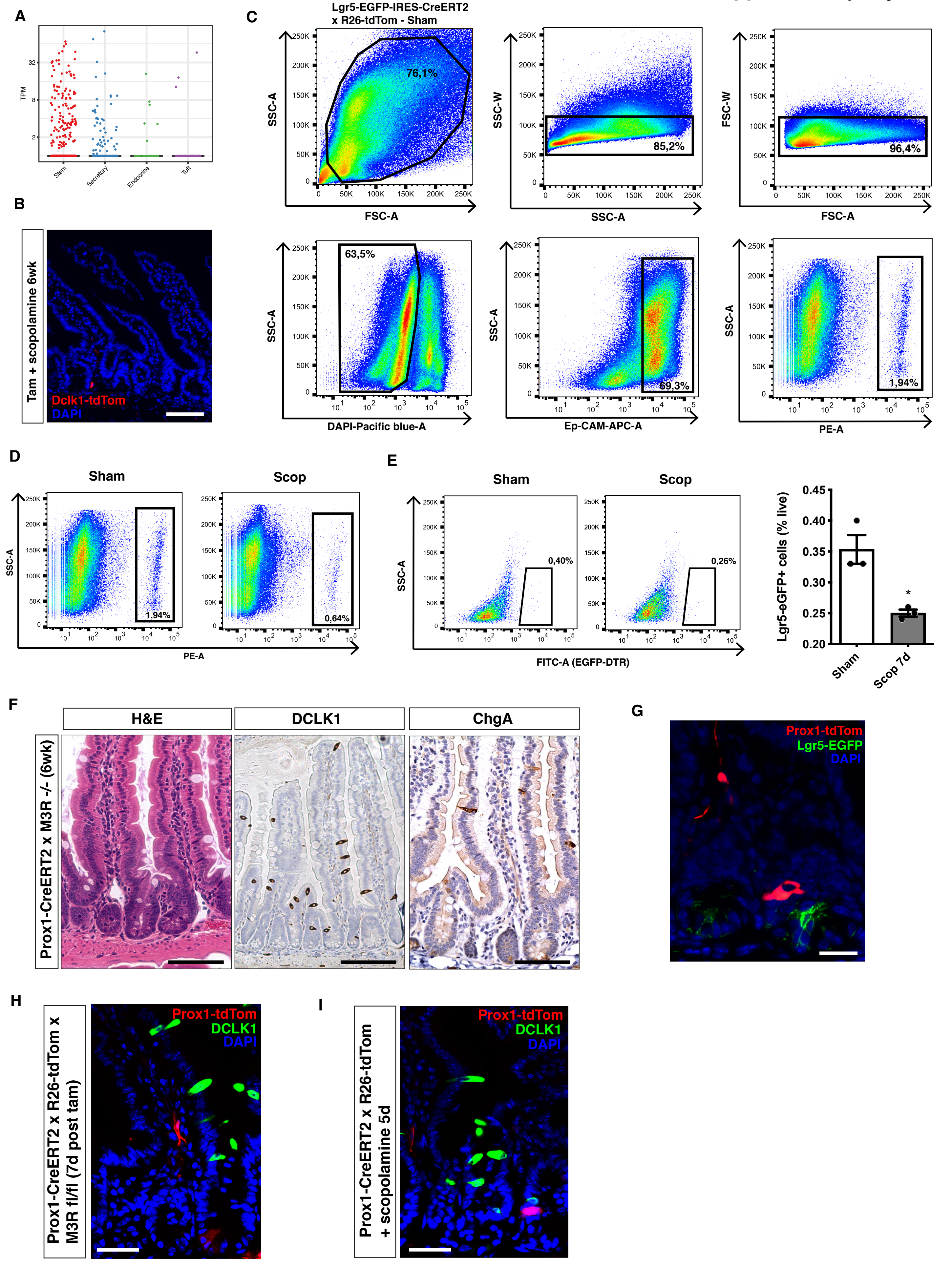

Supplementary Figure 3 related to Fig. 2. Changes in intestinal cholinergic muscarinic transmission differentially affect Lgr5-positive ISC and Prox1-positive endocrine cells. (A) Single-cell *Chrm3* expression among secretory cells, stem cells (defined by *Lgr5*, *Ascl2*, *Axin2* or *Olfm4* expression) and endocrine cell types (expressing *ChgA*, *Tac1*, *Neurog3* or *Prox1*)[1] (TPM = transcript per kilobase million). (B) Long-term (6wk) scopolamine treatment following the induction of Dclk1-BAC-CreERT x R26-tdTom mice (n=6 mice, representative picture, repeated at least 5 times); bar graph = 100 µm. (C, D) Exemplification of flow cytometry gating strategy for sham- and scopolamine-treated induced Lgr5-EGFP-IRES-CreERT2 x R26-tdTom mice (d1 begin scopolamine / sham treatment, d3 Tam induction, d7 sacrifice). (E) Short-term scopolamine treatment (7d) of Lgr5-EGFP-DTR mice revealed significant decrease of Lgr5-EGFP-positive ISC count (n=3 per group; Lgr5-EGFP-DTR Sham Mean = 0.3533, SEM = 0.02333; Lgr5-EGFP-DTR + Scop 7d Mean = 0.25, SEM = 0.005774; unpaired t test, two-tailed, t=4.299, df=4). (F) Analysis of DCLK1-positive tuft cells and ChgA-positive enterochromaffin cells six weeks following single tamoxifen induction of Prox1-CreERT2 x M3R fl/fl mice (n= 3 mice); bar graphs = 100 µm. (G) Analysis of induced Prox1-CreERT2 x R26-tdTom x Lgr5-EGFP-DTR mice revealed the absence of positive cell overlap (representative picture, repeated at least 3 times); bar graph = 50 µm. (H) Conditional M3R ablation in the Prox1-positive cell lineage (Prox1-CreERT2 x R26-tdTom x M3R fl/fl mice, 7d post Tam) did result in DCLK1-positive tuft cell expansion, with absent expansion of Prox1-positive cells or Prox1 tracing of DCLK1-positive tuft cells (n=3 mice, representative picture, repeated at least 5 times); bar graph = 50 µm. (I) Short-term scopolamine treatment (5d) of induced Prox1-CreERT2 x R26-tdTom mice recapitulated a similar phenotype (n=3 mice, representative picture, repeated at least 5

times); bar graph = 50 µm. Source data are provided as a Source Data file. * = $p < 0,05$, SSC = side scatter, FSC = forward scatter, APC = allophycocyanin, PE = phycoerythrin, FITC = fluorescein isothiocyanate.

Supplementary Figure 4

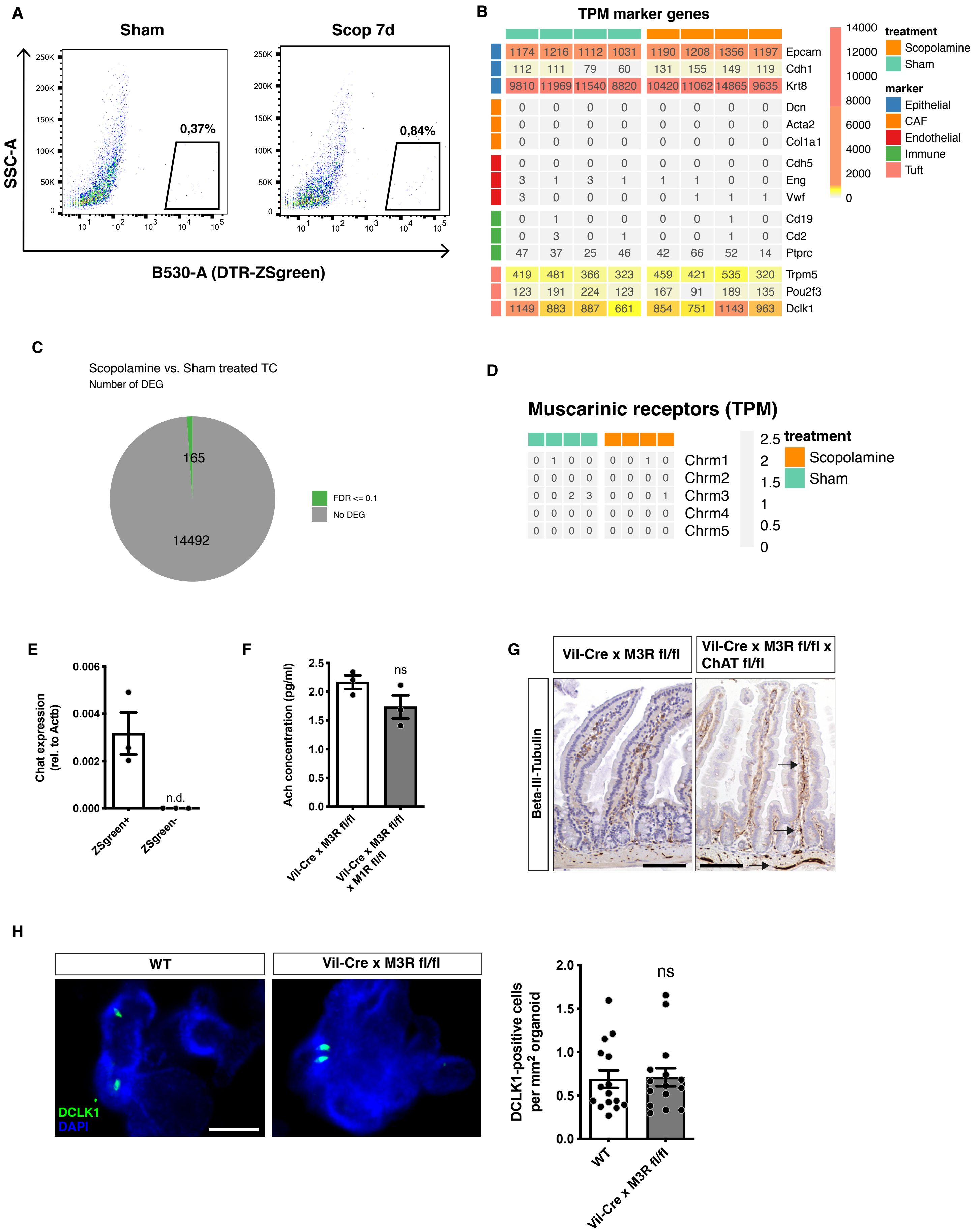

Supplementary Figure 4 related to Figs. 3 and 4. Enteroendocrine tuft cell expansion contributes to cholinergic niche in vivo. (A) Similar gating strategy to Supplementary Fig. 3C showing live/Ep-CAM-positive DTR-ZSgreen-positive tuft cells sorted for bulk RNA sequencing analysis (n=4 mice sham; scopolamine-treated). (B) Examination of gene expression in transcript per kilobase million (TPM) of cell-specific marker genes (epithelial, fibroblasts/CAF, endothelial, immune and tuft cell, respectively) suggests high purity of sorted ZSgreen-positive tuft cells. (C) Pie chart illustrating the number of differentially expressed genes at a false discovery rate of 0.1 in comparison to genes that do not change expression levels significantly. (D) Examination of TPM for muscarinic receptors reveals the preponderant absence of expression among ZSgreen-positive tuft cells. (E) RT-PCR analysis of sorted epithelial (Ep-CAM+) Dclk1-DTR-ZSgreen-positive versus -negative cells confirmed unique expression of *Chat* in tuft cells (n=3 mice); n.d. = not detectable. (F) Simultaneous epithelial ablation of M3R and M1R did not increase mucosal Ach levels compared to Vil-Cre x M3R fl/fl mice alone (n=5 Vil-Cre x M3R fl/fl mice, n=4 Vil-Cre x M3R fl/fl x M1R fl/fl mice; Vil-Cre x M3R fl/fl Mean = 1.958, SEM = 0.143; Vil-Cre x M3R fl/fl x M1R fl/fl Mean = 1.762, SEM = 0.147; unpaired t test, two-tailed, t=0.9458, df=7). (G) Tissue analysis of Vil-Cre x M3R x ChAT fl/fl mice for Beta-III-Tubulin reveals more prominent labeling of stromal nerve fibers compared to Vil-Cre x M3R fl/fl mice (representative pictures, repeated at least 5 times), bar graphs = 100 µm. (H) Analysis of organoid cultures of WT and Vil-Cre x M3R fl/fl mice at day 5 of culture reveals no significant changes to DCLK1-positive tuft cell numbers (n=1 WT and Vil-Cre x M3R fl/fl mouse, values represent 3-4 analyzed organoids per technical replicate, 3-4 technical replicates per sample; WT Mean = 0.69, SEM = 0.102; Vil-Cre x M3R fl/fl Mean = 0.711, SEM = 0.105; unpaired t test, two-tailed, t=0.1464, df=28); bar

graph = 50 µm. ns = not significant, SSC = side scatter. Source data are provided as a Source Data file.

Supplementary Figure 5

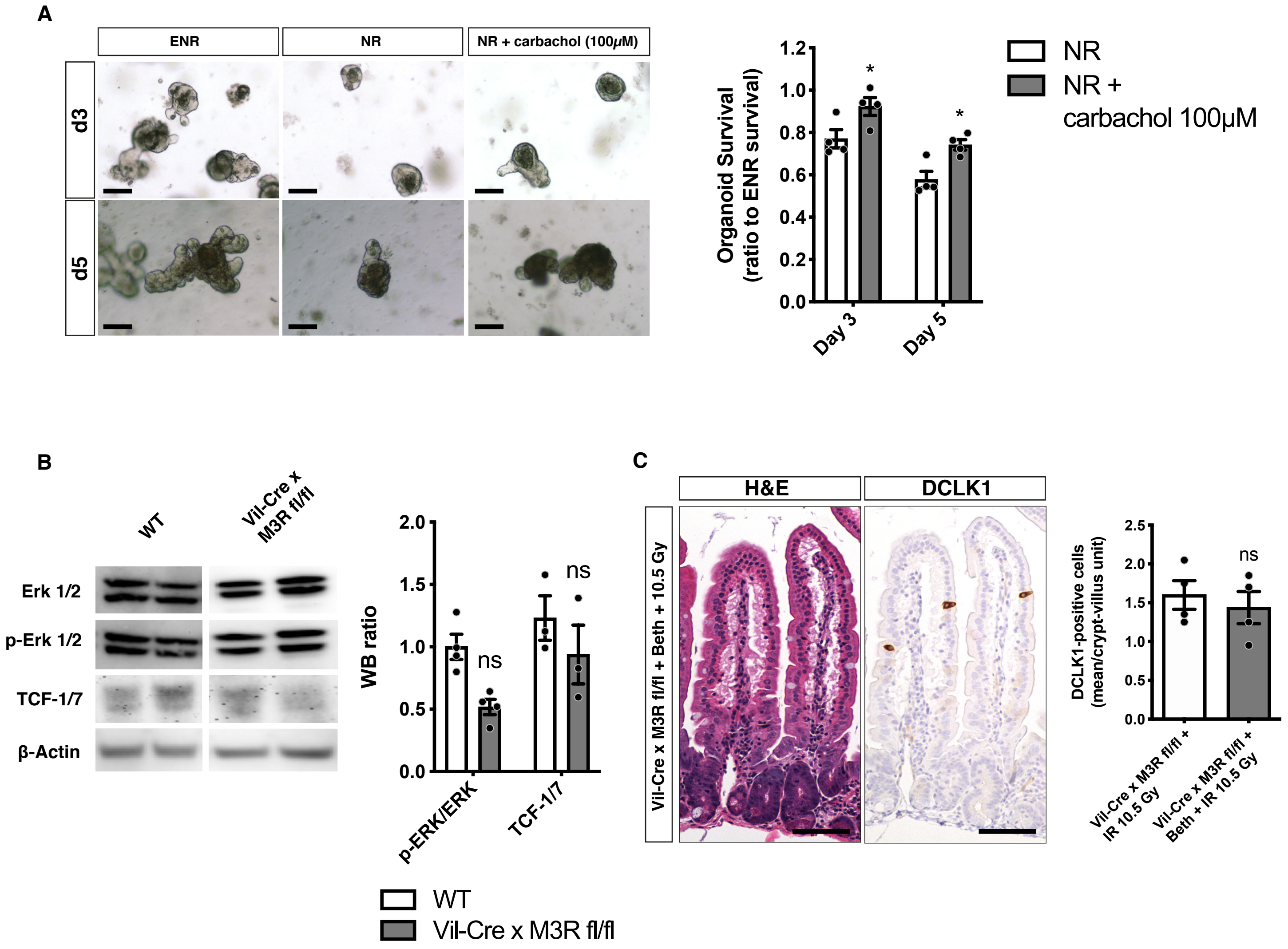

Supplementary Figure 5 related to Figs. 5 and 6. The compensatory increase of cholinergic niche signaling sustains intracellular signaling and homeostasis. (A) Small intestinal organoids treated with the non-selective muscarinic receptor agonist carbachol revealed partial rescue of organoid growth and survival following EGF ligand withdrawal (n=4 mice per group and timepoint; 3 technical replicates per mouse; NR d3 Mean = 0.77, SEM = 0.043, d5 Mean = 0.577, SEM = 0.039; NR + carbachol 100μM d3 Mean = 0.923, SEM = 0.042, d5 Mean = 0.742, SEM = 0.025; ordinary two-way ANOVA, day 3 t=2.834, df=12, day 5 t=3.057, df=12); ENR = EGF, noggin, R-spondin1; d = day; bar graphs = 50 μm. (B) Intracellular ERK activation and TCF did not significantly change in Vil-Cre x M3R fl/fl compared to WT mice (n=4 WT and Vil-Cre x M3R fl/fl mice for p-ERK/ERK; n=3 mice per group for TCF-1/7; WT p-ERK/ERK Mean = 1, SEM = 0.101, TCF-1/7 Mean = 1.231, SEM = 0.178; Vil-Cre x M3R fl/fl p-ERK/ERK Mean = 0.517, SEM = 0.062, TCF-1/7 Mean = 0.938, SEM = 0.236; ordinary two-way ANOVA, p-ERK/ERK t=2.595, df=10, p=0.0527; TCF-1/7 t=1.360, df=10). (C) Short-term Bethanechol treatment (start d1, d3 WBI 10.5Gy IR, d7 analysis) did only moderately decrease DCLK1-positive tuft cell number, while dramatically improving tissue morphology following injury (n=4 per group; Vil-Cre x M3R fl/fl + IR 10.5 Gy Mean = 1.6, SEM = 0.185; Vil-Cre x M3R fl/fl + Beth + IR 10.5 Gy Mean = 1.438, SEM = 0.207; unpaired t test, two-tailed, t=0.5863, df=6). ns = not significant. Source data are provided as a Source Data file.

Supplementary Table 1. List of cDNA primers used for RT-PCR.

Gene ID	NCBI Accession nr	Protein ID	FWD 5´-3´	REV 5´-3´
Hmbs	NM_001110251.1	Porphobilinogen Deaminase	TTGGAAAGACCCTGGAAACC	TGAATTCCTGCAGCTCATCC
Actb	NM_007393.5	Beta-Actin	TAGACTTCGAGCAGGAGATGG	CAGGATTCCATACCCAAGAAGG
Chrm1	NM_001112697.1	Muscarinic acetylcholine receptor M1	CAGAAGTGGTGATCAAGATGCCTAT	GAGCTTTTGGGAGGCTGCTT
Chrm2	NM_203491.3	Muscarinic acetylcholine receptor M2	TGGAGCACAACAAGATCCAGAAT	CCCCTGAACGCAGTTTTCA
Chrm3	NM_033269.4	Muscarinic acetylcholine receptor M3	CTGCGTTCTGACCAAGTGAC	TGTGCAAGGTCATTGTGACTC
Chrm4	NM_007699.2	Muscarinic acetylcholine receptor M4	GTGACTGCCATCGAGATCGTAC	CAAACTTTCGGGCCACATTG
Chrm5	NM_205783.2	Muscarinic acetylcholine receptor M5	TTCCGATTGGTGGTAAAAGC	TTTGGACACTGGGAAGGAAC
Chat	NM_009891.2	Choline acetyltransferase	ACATACCTGATGAGCAACCG	AAAGCTGGAGATGCAGAAGG
Clca1	NM_017474.2	Chloride channel accessory 1	GATCGCTCAGCACTCCAT	GAGCCATTCATCCATTGGTTA
Neurod1	NM_010894.2	Neurogenic differentiation factor 1	ACCAAATCATACAGCGAGAGC	GTCCTCCTCTGCATTCATGG
Hes1	NM_008235.2	Hairy and enhancer of split 1	CATTCTGGAAATGACTGTGAAGC	TGTTAACGCCCTCACACG

Supplementary Table 2. Employed antibodies for immunohistochemistry and immunofluorescence or flow cytometry.

Protein	Company	Cat-nr.	Dilution (IHC[a], IF[b]; FC[c])	Source
DCAMKL1 (DCLK1)	Abcam	31704	1:500 (IHC, IF)	Rabbit
Lysozyme 1	Dako	74097	1:1000 (IHC)	Rabbit
ChgA	Abcam	15160	1:400 (IHC, IF)	Rabbit
M3R	Abcam	126168	1:250 (IF)	Rabbit
PYY	Abcam	22663	1:300 (IHC)	Rabbit
Beta-III-Tubulin	Abcam	18207	1:1000 (IHC)	Rabbit
APC anti-mouse CD326 (Ep-CAM)	Biolegend	118214	1:200 (FC)	
DAPI	BD Pharmingen	564907	1:10000 (FC)	

[a] Immunohistochemistry

[b] Immunofluorescence

[c] Flow cytometry

Supplementary Table 3. Employed antibodies for Western blot (immunoblot).

Target	MW[a]	Company	Lot#	Dilution/Stock	Source
EGFR	180 kDA	Millipore Sigma	06-847	1:500 – 1 mg/ml (=2 µg/ml)	Rabbit
p-EGFR	185 kDA	Millipore Sigma	07-820	1:1000	Rabbit
ERK 1/2	44/42 kDA	Cell Signaling	4695	1:1000	Rabbit
p-ERK	44/42 kDA	Cell Signaling	9102	1:1000	Rabbit
panAkt	60 kDA	Cell Signaling	4691	1:500	Rabbit
p-Akt	60 kDA	Cell Signaling	13038	1:1000	Rabbit
pI3Kinase	110 kDA	Cell Signaling	4249	1:1000	Rabbit
PDK1	68 kDA	Cell Signaling	3062	1:1000	Rabbit
DCAMKL1 (DCLK1)	82 kDA	Abcam	31704	1:2000 – 1 mg/ml (=0.5 µg/ml)	Rabbit
M3R	66 kDA	Abcam	126168	1:500	Rabbit
TCF-1/7	48/50 kDA	Cell Signaling	2203	1:1000	Rabbit
ß-Actin	45 kDA	Cell Signaling	4970	1:5000	Rabbit

[a] Molecular weight

Supplementary References:

1. Haber, A.L. *et al.* A single-cell survey of the small intestinal epithelium. *Nature* **551**, 333-339 (2017).
