# Supplementary material for: Prox1-positive cells monitor and sustain the murine intestinal epithelial cholinergic niche
Source: Nat Commun. 2020 Jan 8;11:111. doi: 10.1038/s41467-019-13850-7 (PMC6949263; doi:10.1038/s41467-019-13850-7)
Supplement: Supplementary file 3 — Description of Additional Supplementary Files [file 41467_2019_13850_MOESM3_ESM.pdf]

Title: Supplementary Data 1

Description: Gene set enrichment analysis (GSEA) of consensus signature genes for endocrine epithelial cell types from single-cell datasets (Refs. Haber et al., Nature 2017; Gehart et al., Cell 2019) in the gene expression signature between scopolamine vs. sham-treated ZSgreen-positive tuft cells.
