# Supplementary material for: Prox1-positive cells monitor and sustain the murine intestinal epithelial cholinergic niche
Source: Nat Commun. 2020 Jan 8;11:111. doi: 10.1038/s41467-019-13850-7 (PMC6949263; doi:10.1038/s41467-019-13850-7)
Supplement: Supplementary file 4 — Reporting Summary [file 41467_2019_13850_MOESM4_ESM.pdf]

nature research

Corresponding author(s): Timothy C. Wang

Last updated by author(s): 2019/11/14

Reporting Summary

Nature Research wishes to improve the reproducibility of the work that we publish. This form provides structure for consistency and transparency in reporting. For further information on Nature Research policies, see Authors & Referees and the Editorial Policy Checklist.

Statistics

For all statistical analyses, confirm that the following items are present in the figure legend, table legend, main text, or Methods section.

n/a	Confirmed	
☐	☒	The exact sample size (*n*) for each experimental group/condition, given as a discrete number and unit of measurement
☐	☒	A statement on whether measurements were taken from distinct samples or whether the same sample was measured repeatedly
☐	☒	The statistical test(s) used AND whether they are one- or two-sided<br>*Only common tests should be described solely by name; describe more complex techniques in the Methods section.*
☒	☐	A description of all covariates tested
☐	☒	A description of any assumptions or corrections, such as tests of normality and adjustment for multiple comparisons
☐	☒	A full description of the statistical parameters including central tendency (e.g. means) or other basic estimates (e.g. regression coefficient) AND variation (e.g. standard deviation) or associated estimates of uncertainty (e.g. confidence intervals)
☐	☒	For null hypothesis testing, the test statistic (e.g. *F*, *t*, *r*) with confidence intervals, effect sizes, degrees of freedom and *P* value noted<br>*Give P values as exact values whenever suitable.*
☒	☐	For Bayesian analysis, information on the choice of priors and Markov chain Monte Carlo settings
☒	☐	For hierarchical and complex designs, identification of the appropriate level for tests and full reporting of outcomes
☒	☐	Estimates of effect sizes (e.g. Cohen's *d*, Pearson's *r*), indicating how they were calculated

Our web collection on statistics for biologists contains articles on many of the points above.

Software and code

Policy information about availability of computer code

Data collection	Data analyzed in the present manuscript originates from bulk RNA sequencing and data processed at Columbia Genome Center. Further sources of data and software for data processing are indicated below.
Data analysis	Sequencing data was processed using RTA (Illumina), bcl2fastq2 (version 2.20), coupled with adaptor trimming. Pseudoalignment was carried out to a kallisto index created from the murine GRCm38 transcriptome using kallisto (0.44.0). Estimated counts and transcripts per kilobase million (TPM) per gene were computed from the kallisto output using the tximport R package (Soneson et al., 2015). A negative binomial generalized linear model was used to test for differentially expressed genes between Sham and Scopolamine treated tuft cells as implemented in the DESeq2 R package (Love et al, 2014). Expression in TPM for select genes was illustrated in a heatmap using the pheatmap R package (Kolde; https://CRAN.R-project.org/package=pheatmap, 2019). Consensus signature genes for subsets of epithelial cells were retrieved from the supplement of Haber et al. (Nature, 2017) and Gehart et al. (Cell, 2019) and their enrichment in the gene expression signature between Scopolamine and Sham treated tuft cells was examined using methodology as described by Subramanian et al. (PNAS, 2005). Select enrichment results were illustrated using custom R code. A tuft cell regulatory network was reverse engineered by ARACNe-AP (Lachmann et al, 2016) using 102 single-cell RNA-Seq gene expression profiles of the full length dataset from Haber et al. Genes with less than one transcript per kilobase million in at least 10 of the 102 tuft cells were removed. ARACNe was run with standard settings (using data processing inequality (DPI), with 100 bootstrap iterations using all gene symbols mapping to a set of 1813 transcription factors that includes genes annotated in the Gene Ontology (GO) molecular function database as GO:0003700 (transcription factor activity), GO:0004677 (DNA binding), GO:0030528 (transcription regulator activity) or as GO:0004677/ GO: 0045449 (regulation of transcription). Thresholds for the tolerated DPI and mutual information P value were set to 0 and 10–8, respectively. Master regulator analysis was performed by interrogating the gene expression signature between scopolamine and sham treated tuft cells using the tuft cell regulatory network and the msviper algorithm as implemented in the viper R package (Alvarez, 2016; Alvarez, 2014).

For manuscripts utilizing custom algorithms or software that are central to the research but not yet described in published literature, software must be made available to editors/reviewers. We strongly encourage code deposition in a community repository (e.g. GitHub). See the Nature Research guidelines for submitting code & software for further information.

Data

Policy information about availability of data

All manuscripts must include a data availability statement. This statement should provide the following information, where applicable:
- Accession codes, unique identifiers, or web links for publicly available datasets
- A list of figures that have associated raw data
- A description of any restrictions on data availability

All relevant data are available from the authors upon request. The RNA sequencing data reported in this study are available from Gene Expression Omnibus with accession code GSE138365 [https://www.ncbi.nlm.nih.gov/geo/query/acc.cgi?acc=GSE138365]. The source data underlying Fig. 1B-D, Fig. 2C-E, Fig. 3, Fig. 4B-E, Fig. 5B-D, Fig. 6A-D, Supplementary Fig. 1A, B, D, E, Supplementary Fig. 2A-E, Supplementary Fig. 3E, Supplementary Fig. 4E, F, H and Supplementary Fig. 5A-C are provided as a Source Data file.

Field-specific reporting

Please select the one below that is the best fit for your research. If you are not sure, read the appropriate sections before making your selection.

☒ Life sciences ☐ Behavioural & social sciences ☐ Ecological, evolutionary & environmental sciences

For a reference copy of the document with all sections, see nature.com/documents/nr-reporting-summary-flat.pdf

Life sciences study design

All studies must disclose on these points even when the disclosure is negative.

Sample size	No statistical methods were used to predetermine sample size. Mouse experiments were performed on at least n = 3 biological replicates, on average including 4-5 mice per group and experiment.
Data exclusions	Data was only excluded from the analysis in cases of outliers. Full data is provided in the Source Data file.
Replication	All attempts at replication were successful. All graphs represent data with at least three biological replicates, all images represent findings reproduced at least twice in the laboratory. Where applicable, technical replicates have been indicated in the respective Method section or Figure legend.
Randomization	Animal experiments were performed on littermates randomly allocated to different experimental groups.
Blinding	The investigators were blinded for image and quantitative analysis.

Reporting for specific materials, systems and methods

We require information from authors about some types of materials, experimental systems and methods used in many studies. Here, indicate whether each material, system or method listed is relevant to your study. If you are not sure if a list item applies to your research, read the appropriate section before selecting a response.

Materials & experimental systems

n/a	Involved in the study	
☐	☒	Antibodies
☒	☐	Eukaryotic cell lines
☒	☐	Palaeontology
☐	☒	Animals and other organisms
☒	☐	Human research participants
☒	☐	Clinical data

Methods

n/a	Involved in the study	
☒	☐	ChIP-seq
☐	☒	Flow cytometry
☒	☐	MRI-based neuroimaging

Antibodies

Antibodies used	Immunohistochemistry, immunofluorescence, flow cytometry:<br>DCAMKL1 (DCLK1), company Abcam, cat-nr. 31704<br>Lysozyme 1, company Dako, cat-nr. 74097<br>ChgA, company Abcam, cat-nr. 15160<br>M3R, company Abcam, cat-nr. 126168<br>PYY, company Abcam, cat-nr. 22663<br>Beta-III-Tubulin, company Abcam, cat-nr. 18207<br>APC anti-mouse CD326 (Ep-CAM), company Biolegend, cat-nr. 118214<br>DAPI, company BD Pharmingen, cat-nr. 564907<br><br>Western blot (immunoblot):<br>EGFR, company Millipore Sigma, cat-nr. 06-847<br>p-EGFR, company Millipore Sigma, cat-nr. 07-820<br>ERK 1/2, company Cell Signaling, cat-nr. 4695<br>p-ERK, company Cell Signaling, cat-nr. 9102<br>panAkt, company Cell Signaling, cat-nr. 4691<br>p-Akt, company Cell Signaling, cat-nr. 13038<br>pI3Kinase, company Cell Signaling, cat-nr. 4249<br>PDK1, company Cell Signaling, cat-nr. 3062<br>DCAMKL1 (DCLK1), company Abcam, cat-nr. 31704<br>M3R, company Abcam, cat-nr. 126168<br>TCF-1/7, company Cell Signaling, cat-nr. 2203<br>ß-Actin, company Cell Signaling, cat-nr. 4970
Validation	Antibodies for mouse studies were validated by comparison of positive and negative controls and positive results were verified with the manufacturer´s information and referenced publications.

Animals and other organisms

Policy information about studies involving animals; ARRIVE guidelines recommended for reporting animal research

Laboratory animals	All studies were performed on adult C57BL/6J mice or genetically engineered mouse models as indicated in the Methods section; only adult mice aged 6-10 weeks at the time of sacrifice or tamoxifen induction were analyzed, both sexes were used.
Wild animals	The study did not involve wild animals.
Field-collected samples	The study did not involve samples collected from the field.
Ethics oversight	All mouse studies were approved by the Columbia University Institutional Animal Care and Use Committee (directed by Mary Jo Shepherd, Execute Director of the Office of the IACUC, Columbia University).

Note that full information on the approval of the study protocol must also be provided in the manuscript.

Flow Cytometry

Plots

Confirm that:

☒ The axis labels state the marker and fluorochrome used (e.g. CD4-FITC).

☒ The axis scales are clearly visible. Include numbers along axes only for bottom left plot of group (a 'group' is an analysis of identical markers).

☒ All plots are contour plots with outliers or pseudocolor plots.

☒ A numerical value for number of cells or percentage (with statistics) is provided.

Methodology

Sample preparation	Small intestinal crypts were isolated from proximal murine jejunum (Haber et al., 2017). Harvested pieces were minced, washed in ice-cold DPBS, subjected to EDTA-chelation (10 mM solution, exchanged three times) and mild shaking at 4°C. An additional step of mild pipetting using DPBS/5% FBS solution following EDTA chelation was used to increase crypt cell yield. Single-cell suspensions were then obtained from enriched crypts and villi by incubation in culture media containing ROCK inhibitor for 45 min at 37°C, followed by mild mechanical dissociation using a syringe with a 21 G needle (Sato et al, 2009). The suspension was then filtered through a 40 µm mesh and dissociation and cell count determined using a hemocytometer. Stainings were performed with fluorophore-conjugated antibodies in 2% FBS/PBS solution for 25 min on ice in the dark. Live/dead staining has been achieved by addition of DAPI right before data acquisition.
Instrument	BD Fortessa flow cytometer (analysis), FACS Aria II (sorting)
Software	FACS Diva Software and FlowJo V10 have been employed to process and analyze data.
Cell population abundance	Post-sort fraction analysis has not been performed; RT-PCR and sequencing analysis of the respective sorted cell populations has been performed to ensure specificity and purity of sorting.
Gating strategy	FSC/SSC gating was employed to enrich subsequent gates for single cells and exclude doublets from analysis; subsequent live/ dead separation was achieved by addition of DAPI prior to analysis or sorting (cells below 10`3 were considered viable); Ep-CAM-APC gating included cells 10`3 or higher (Ep-Cam-high), the reporter-positive cell population (PE-A / FITC-A / B530-A-positive) was determined by simultaneous analysis of negative controls and sorting of positive and negative populations for RT-PCR analysis. Color compensation controls were used for the initial setup of gating strategy. An exemplifying gating strategy is included in Supplementary Fig. 3C.

☒ Tick this box to confirm that a figure exemplifying the gating strategy is provided in the Supplementary Information.
